# Light-driven secondary structural remodeling in biomimetic nanosystem to enhance tumor chemo-phototherapy

**DOI:** 10.1016/j.mtbio.2025.101955

**Published:** 2025-06-06

**Authors:** Weijie Wang, Chenguang Sun, Linhao Jing, Yaning Xia, Shuijun Zhang, Yupeng Shi

**Affiliations:** a Department of Hepatobiliary and Pancreatic Surgery, The First Affiliated Hospital of Zhengzhou University, Zhengzhou, 450052, China; b Department of MRI, The First Affiliated Hospital of Zhengzhou University, Zhengzhou, 450052, China

**Keywords:** Drug delivery, In situ assembly, Hepatocellular carcinoma, Photothermal therapy, Homologous targeting

## Abstract

The integration of chemotherapy and phototherapy for treating advanced liver cancer has gained considerable attention. However, challenges such as short drug retention times significantly impact patient prognosis. We introduce a light-triggered nanosystem that employs molecular aggregation control for PTT and sustained chemotherapy. This nanosystem, known as Reg/IR783@CM nanoparticles (RIMNPs), consists of a core-shell carrier-free nanodrug self-assembled from the chemotherapy drug regorafenib (Reg) and the photothermal agent IR783, coated with a homologous liver cancer cell membrane. The developed core-shell nanocarrier exhibits excellent water dispersibility, high drug load, extended blood circulation, and tumor site enrichment. Upon light exposure, the nanosystem provides outstanding near-infrared imaging and robust photothermal effects. Concurrently, light exposure accelerates the degradation of the outer IR783 layer, resulting in regorafenib exposure and triggering secondary assembly, which significantly enhances drug retention at the tumor site. Our findings indicate that the nanosystem effectively suppresses tumor growth by combining photothermal therapy with the inhibition of tumor cell proliferation and angiogenesis, and by modulating tumor-associated macrophages. Notably, this nanosystem also demonstrates low cytotoxicity and high biocompatibility. This study presents a novel light-driven in-situ assembly strategy, offering a simplified and effective approach for constructing tumor imaging and treatment systems.

## Graphical abstract

**Figure:**
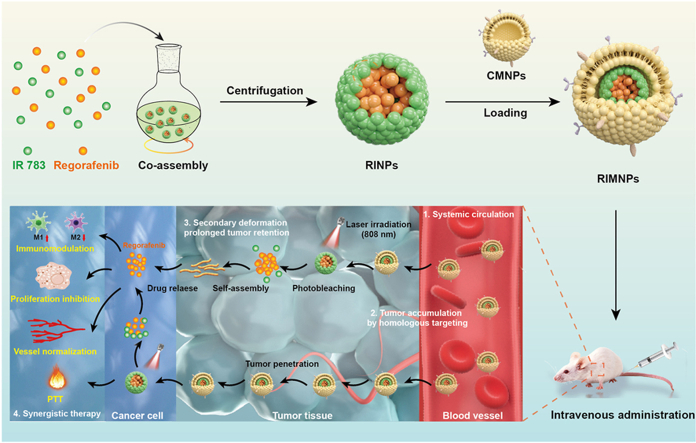

## 1 Introduction

Hepatocellular carcinoma (HCC) is the leading cause of cancer-related deaths globally, with a distressingly low five-year survival rate of only 3 % [1,2]. Alarmingly, over half of HCC patients are diagnosed at an advanced or incurable stage, rendering systemic therapy the sole option to enhance their overall survival [3]. Due to its highly vascular nature, antiangiogenic tyrosine kinase inhibitors (TKIs) that target the vascular endothelial growth factor (VEGF) pathway have emerged as the most effective treatment for patients with advanced HCC (aHCC) [4]. Regorafenib (Reg), a broad-spectrum kinase inhibitor, not only counters molecular escape pathways from VEGF inhibition but also bolsters anti-tumor immunity by reducing the infiltration of tumor-associated macrophages (TAMs) and altering TAM polarization [5,6]. Presently, Reg is approved for second-line treatment of aHCC, refractory colorectal cancer, and gastrointestinal stromal tumors [7]. Nonetheless, the poor drug characteristics of Reg, including solubility, dissolution, permeability, circulation, and distribution, often limit the recommended dosage due to severe treatment-related side effects, such as hand-foot skin reactions, diarrhea, and hypertension, thus constraining its effectiveness [8,9].

In recent years, significant advancements in nanotechnology and tumor biology have propelled the nanomedicine field forward. Drug delivery systems based on nanomaterials can enhance the dissolution and delivery efficiency of traditional drugs, increase their bioavailability, and mitigate adverse reactions, making them a subject of extensive discussion [[10], [11], [12], [13]]. However, the efficacy, safety, and pharmacokinetics of engineered nanocarriers still require systematic study. Furthermore, several challenges hinder the large-scale production of nanocarrier drugs, including low drug load, complex manufacturing processes, and potential systemic toxicity and immunogenicity, significantly limiting their clinical application [14,15]. Fortunately, the innovative concept of carrier-free nanodrugs has been introduced, utilizing nanostructures that form spontaneously or through triggered self-assembly of drug molecules via non-covalent bonds such as hydrogen bonding, hydrophobic interactions, and π-π stacking [[16], [17], [18]]. These carrier-free nanodrugs, with their high drug-loading capacity, absence of inert carrier materials, and simple, eco-friendly preparation methods, hold substantial promise for clinical translation [19,20]. Nevertheless, the pre-self-assembly strategy is constrained by inadequate tumor penetration and limited blood circulation [21]. In contrast, cancer diagnosis and treatment leveraging in-situ self-assembly offer numerous benefits. These include enhanced blood circulation of monomers, sustained drug delivery dynamics, reduced drug resistance, and the capacity to target deep tumors and organelles, thus boosting the therapeutic effectiveness [22]. In-situ self-assembly is activated by various stimuli, including endogenous factors like enzymes, pH, and redox molecules, as well as exogenous ones such as light, magnetic fields, and ultrasound [23,24]. Among them, optical control, with its high spatiotemporal resolution and precise regulation ability, can reversibly adjust the physical and chemical states of photo-responsive materials (such as azobenzene and spiropyranan) through light switches, thereby achieving dynamic control of the secondary deformation of nanoparticles [25,26]. Jiang et al. utilized the self-assembly of polypeptide molecules to form spherical nanoparticles, which could be transformed into rod-shaped particles under the action of exogenous light, thereby enhancing the accumulation of nanomedicines within tumors [27]. Therefore, the adaptability and dynamic switching capability of self-assembled nanomaterials are becoming increasingly vital in bioimaging and tumor therapy.

Photothermal therapy represents a new, efficient, selective, minimally invasive, and rapid recovery tumor treatment method with a low risk of complications, showing significant promise for in-situ liver cancer treatment [28,29]. High-performance photothermal reagents are an important link in their photothermal therapy. So far, a variety of material systems have been developed. Among them, it mainly includes gold nanorods, copper sulfide (CuS), graphene, polypyrrole, polydopamine, indoleine green, Prussian blue, porphyrin, *etc*. However, the use of intense lasers, which can raise local temperatures to 50 °C, may harm normal organs adjacent to the tumor while targeting the cancerous cells [30]. It is crucial to manage the treatment temperature and duration to avoid damaging healthy tissues. IR783 is a near-infrared cyanine-based small-molecule dye characterized by high photothermal conversion efficiency and excellent biocompatibility, making it widely utilized as a photothermal agent. Furthermore, studies have demonstrated that IR783 can actively target and accumulate in tumor cells through organic anion transporter peptides (OATPs), which are highly expressed in tumors. This tumor-targeting mechanism has garnered significant attention for its application in photothermal therapy of cancer [31,32]. Moreover, some research indicates that IR783 can spontaneously assemble with various hydrophobic small molecule drugs to create stable nanoparticles, enhancing the solubility and bioavailability of anticancer drugs while exerting synergistic antitumor effects through the nanoparticles' optical properties [33,34]. However, like other tricarbonyl cyanine dyes, IR783 is prone to degradation under near-infrared light exposure, which considerably limits its biomedical research and applications. Thus, harnessing its photodegradation properties constructively for tumor diagnosis and treatment could be highly significant. The effectiveness of any treatment hinges on the targeted accumulation of drugs at the cancer site. Recently, biomimetic nanoparticles coated with active cell membranes have emerged as a new type of nanocarrier. In 2011, Zhang's research group first reported this technology and achieved effective drug delivery by encapsulating nanoparticles with red blood cell membranes, while demonstrating longer retention time and immune escape function [35]. At present, the main sources of natural cell membranes include red blood cell membranes, platelet membranes, white blood cell membranes, cancer cell membranes, stem cell membranes and their hybrid membranes [36,37]. These nanoparticles encapsulated in the cell membrane integrate the advantages of protocell and core nanoparticles which exhibit many characteristics of their source cells, including enhanced biocompatibility, immune evasion, prolonged blood circulation and targeted tumor delivery, presenting significant potential for targeted drug delivery and effective cancer treatment [38,39].

**Scheme 1:**
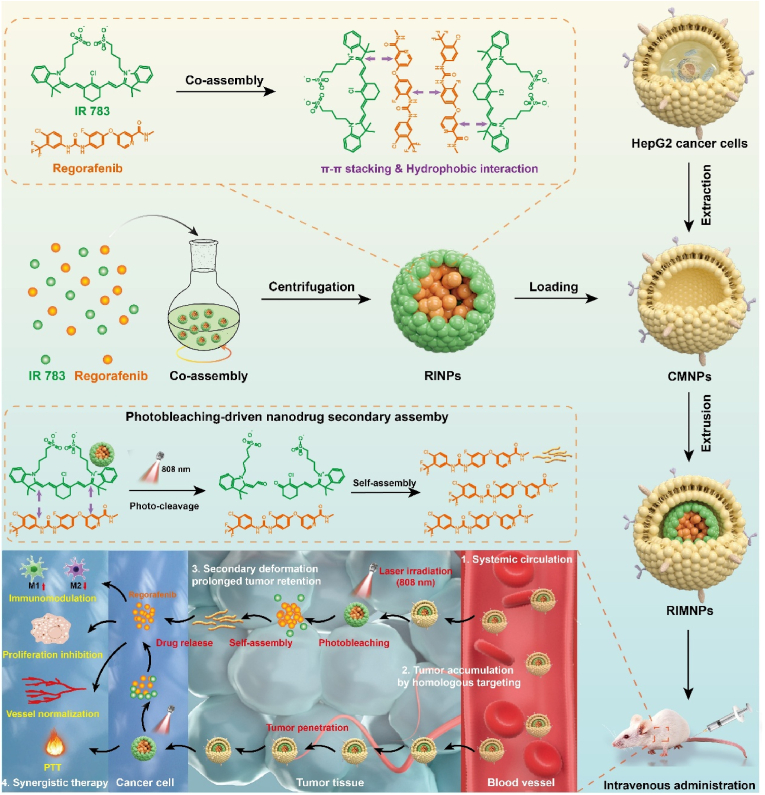
Schematic illustration of preparing cancer membrane-coated carrier-free nanosystem (abbreviated as RIMNPs) and the synergistic photothermal-chemotherapy and enhanced immunotherapy against hepatocellular carcinoma.

In this study, IR783 and regorafenib were utilized as raw materials to synthesize carrier-free nanoparticles (Reg/IR783 nanoparticles, RINPs) through self-assembly. Subsequently, intelligent biomimetic nanoparticles were crafted by coating these with liver cancer cell membranes (Scheme 1). The resulting nanosystem (Reg/IR783@CM nanoparticles, RIMNPs) overcomes the challenges of poor solubility and low bioavailability associated with hydrophobic Reg in circulation while demonstrating excellent reproducibility and colloidal stability. Moreover, organic molecules engage in functional self-assembly without any superfluous components, enhancing the nanomedicine's biocompatibility and biosafety. Due to the membrane proteins of cancer cells possessing homologous binding capabilities, the engineered biomimetic nanomedicine (RIMNPs) effectively targets and accumulates in tumor nests, minimizing loss during in vivo circulation. Upon reaching the liver tumor site, precise near-infrared imaging-guided laser irradiation induces local hyperthermia, directly annihilating tumor cells. Notably, IR783 absorbs heat, undergoes degradation, releases regorafenib, and subsequently reassembles into rod-shaped nanomaterials. This process significantly extends its presence at the tumor site, thereby facilitating a synergistic therapeutic effect combining photothermal therapy (PTT) and extended-duration chemotherapy.

## 2 Materials and methods

### 2.1 Materials

Regorafenib (99.9 %) was obtained from Shanghai Tao Technology Biotechnology Co., Ltd. IR783 was sourced from Shanghai Aladdin Biochemical Technology Co., Ltd. Dulbeccos modified eagle medium (DMEM) and fetal bovine serum (FBS) was procured from Beijing Solarbio Technology Co., Ltd. The following reagents were acquired from Beyotime: ethylenediaminetetraacetic acid (EDTA), trypsin, 4’,6-diamidino-2-phenylindole dihydrochloride (DAPI), propidium iodide (PI), cell counting kit-8 (CCK-8), hematoxylin-eosin (HE) staining kit, membrane protein extraction kit, and TdT-mediated dUTP Nick-End Labeling (TUNEL) apoptosis assay kit. The polycarbonate membrane (200 nm), anti-N-cadherin antibody, and secondary antibodies, including goat anti-mouse IgG and goat anti-rabbit IgG, were obtained from Wuhan Servicebio Biotechnology Co., Ltd. The following antibodies were procured from Abcam: anti-EpCAM, anti-Galectin-3, anti-Ki67, anti-CD31, anti-alpha-SMA, anti-F4/80, anti-CD80, and anti-CD206. All materials and reagents were used as received unless otherwise specified.

### 2.2 Screening of co-assembled nanoparticles

The nanoassemblies were prepared using the one-step co-assembly method [40]. Typically, regorafenib (40 μL, 5 mg/mL, in DMSO) is added by drops to a set of 60 μL aqueous solutions containing gradient concentrations IR783 (10, 50, 100, 200, 500, and 1000 μg/mL). Then the particle size and PDI of each solution were determined by DLS.

### 2.3 Synthesis of Reg/IR783 nanoparticles (RINPs)

Initially, regorafenib (400 μL, 5 mg/mL, in DMSO) was added dropwise to a group of 600 μL aqueous solutions containing concentrations of IR783 (500 μg/mL) under vigorous vortex. The mixture was then stirred at room temperature for 30 min. The resulting self-assembled suspension was centrifugated (20000 g) for 30 min to obtain the precipitate. Finally, the precipitate underwent three washes with distilled water to acquire Reg/IR783 nanoparticles (RINPs). The contents of IR783 and Reg in the resulting nanoparticle solution were determined by HPLC analysis. Their loading capacity and encapsulation efficiency were calculated as follows:

$$\text{Loading}\;\text{capacity}\;\left(\%\right)=\frac{W_{IR783}\;or\;W_{Reg}}{W_{RINPs}}\times 100\%$$

$$\text{Encapsulation}\;\text{efficiency}\;\left(\%\right)=\frac{W_{IR783}\;or\;W_{Reg}}{W_{feeding\;IR783\;}\;or\;W_{feeding\;Reg}}\times 100\%$$

### 2.4 Synthesis of Reg/IR783@CM nanoparticles (RIMNPs)

The HepG2 Cell membrane extraction followed the procedure of our previous study [41]. Subsequently, they underwent sonication for 1 min and were then extruded 15 times through polycarbonate porous membranes with pore sizes of 800 nm, 400 nm, and 200 nm, respectively, using an Avanti mini extruder (Avanti Polar Lipids, USA). The HepG2 CCM-derived vehicles (CMNPs) were coated onto RINPs cores by co-extruding vehicles and cores through a 220 nm polycarbonate membrane to form RIMNPs. For mass production, the CMNPs (1.0 mL) were combined with RINPs by sonication using gradient programs (50 W, 5 min, 5s on/off cycles). Subsequently, the resulting multicomponent mixed system containing RIMNPs was centrifuged at 1000 rpm for 10 min to remove excess CMNPs.

### 2.5 Characterization of RIMNPs

The Ultraviolet–visible (UV) spectrometer was used to determine the spectra of Reg, IR783, and RINPs. Transmission electron microscopy (TEM) was employed to analyze the morphology of CMNPs, RINPs, and RIMNPs. Particle size and zeta potential were assessed using a Malvern Nano-ZS analyzer. The sodium dodecyl sulfate-polyacrylamide gel electrophoresis (SDS-PAGE) and western blot (WB) analysis was utilized to characterize the cancer cell membrane protein of RIMNPs. The stability of RIMNPs NPs was measured of particle size and PDI by DLS within a total period of 5 days.

### 2.6 The photothermal properties of RIMNPs

The photothermal properties of RIMNPs were characterized by an 808 nm laser irradiation. Briefly, to investigate the concentration-dependence of the photothermal effect, virous concentrations of RIMNPs (0, 25, 50, 100 μg/mL) were irradiated for 5 min with 1.0 W/cm^2^. Moreover, the power-dependence of the photothermal effect was illustrated by interacting with 50 μg/mL RIMNPs with intensities of 0.5 W/cm^2^, 1.0 W/cm^2^, and 1.5 W/cm^2^ respectively. The photothermal stability of RIMNPs was evaluated by irradiated RIMNPs solution (50 μg mL^−1^) in 5 repeated cycles of 10 min irradiation ON and 5 min OFF at power densities of 1.0 W cm^−2^. An IR thermal camera (Testo 865, Germany) was used to monitor the changes in solution temperature during the irradiation.

### 2.7 Drug release in vitro

*In vitro* Reg release from the RIMNPs was studied in PBS buffer with different conditions. For each release study, 1 mL of RIMNPs (1 mg mL^−1^) were dispersed inside a dialysis bag (MWCO = 8000 Da) that was soaked in 19 mL different buffer mediums (pH 7.4 and pH 5.5) with or without exposure to 808 nm laser irradiation (1.0 W cm^−2^) for 10 min with gentle shaking at 37 °C. At selected time intervals, the sample was collected and 2 mL of solution outside the dialysis bag was removed. Then, 2 mL of fresh PBS buffer was added. The removed solution was properly diluted, and the amount of Reg molecules present was measured by the curve of UV–vis spectroscopy.

### 2.8 Cell uptake and targeted studies

HepG2, LO2 and HUVECs cells were cultured in DMEM supplemented with 10 % FBS and 100 units/mL antibiotics (Penicillin-Streptomycin) at 37 °C in a 5 % CO_2_ humidified atmosphere. HUVECs cells were cultured in DMEM supplemented with 10 % FBS and 100 units/mL antibiotics (Penicillin-Streptomycin) at 37 °C in a 5 % CO_2_ humidified atmosphere. To study the endocytosis behavior of RIMNPs, HepG2 cells were seeded onto a 60 mm culture plate at 10^5^ cells/plate density and incubated for 24 h. Then, the culture medium was removed and replaced with a specific concentration of RIMNPs. After co-culture with RIMNPs for 0, 2, 4, 6 h, respectively, the HepG2 was collected for flow cytometry analysis. Moreover, bio-TEM of HepG2 cells were conducted after incubation with RIMNPs for 6 h and irradiation with an 808 nm laser for 5 min. To study cell targeting, RINPs and RIMNPs were co-cultured with HepG2 or LO2 for 12 h, respectively. The cells were washed with PBS three times, fixed with 4 % (v/v) paraformaldehyde for 2 h, and stained the nucleus with DAPI in PBS buffer for 15 min. Finally, cell imaging was performed using fluorescence microscopy. The assessment of immune evasion capability involved the uptake of RINPs and RIMNPs by macrophage RAW 264.7, which was performed by the description above.

### 2.9 In vitro anti-tumor studies

The CCK-8 assay was utilized to evaluate cell viability. HepG2 cells were individually seeded at a density of 5000 cells per well in 96-well plates, followed by overnight incubation. After dilution with 200 μL of the medium, different concentrations of free Reg, RINPs, RIMNPs, and RIMNPs + Laser groups (containing reg 0.5, 2, 4, 8, and 10 μg/mL) were utilized to replace the original culture medium. The treatment for the RIMNPs + Laser group was identical to that of the other groups except that the cells were exposed to a near-infrared laser at a wavelength of 808 nm with a power density of 2.0 W/cm^2^ for a duration of 5 min following an incubation period of 6 h. All cells were incubated for 24 h with five triplicate wells per concentration. After removing the medium, each well was supplemented with 100 μL CCK-8/medium (1:10), and the absorbance at 450 nm was measured using a microplate reader.

The apoptosis of cancer cells was assessed using flow cytometry. HepG2 cells were seeded at a density of 1 × 10^5^ per well in 6-well plates and incubated overnight to allow for adherence and growth. The cultures were subsequently incubated with Reg, RINPs, RIMNPs, and RIMNPs Laser (with a concentration of Reg as 8 μg/mL) in a 24-well plate for 24 h. The previous culture medium was aspirated, and tryptase (without EDTA) was added. The cells were then centrifuged with the previous culture medium, and 5 × 10^4^ cells were resuspended. Subsequently, the cells were stained using a PI and Annexin V-FITC kit, followed by apoptosis detection through flow cytometry.

Furthermore, a live/dead viability assay was also conducted to assess the efficacy of various treatments on cancer cells further. The eliminated HepG2 cells were resuspended in the medium, counted, and subsequently inoculated into a 24-well plate at a density of 1 × 10^4^ cells per well. Three parallel wells were placed in each group and incubated overnight in a cell incubator at 37 °C with 5 % CO_2_. After the cells reached a 70–80 % growth rate under the microscope, Reg, RINPs, RIMNPs, and RIMNPs Laser (with a concentration of 8 μg/mL for Reg) were introduced and incubated with the cells for 24 h. Remove the previous culture medium and perform two rinses with PBS. Subsequently, live and dead cells were stained using the Calcein-AM/PI kit. After being incubated in a 37 °C incubator for 30 min, the fluorescence images were captured using an inverted fluorescence microscope and quantified using Image J software.

### 2.10 Animals and tumor model

The BALB/cA-nu mice (∼15 g) were subcutaneously inoculated with HepG2 cells (1 × 10^7^) in the flank region to establish a subcutaneous tumor xenograft model. Once the tumor volume reached approximately 50 mm^3^, it was utilized for subsequent in vivo experiments in mice. Additionally, hemolysis experiments were conducted using female BALB/cA-nu mice aged 4–6 weeks. All animals received care following the Guidance Suggestions for the Care and Use of Laboratory Animals. The procedures were approved by the Ethics Committee of Zhengzhou University's Academy of Medical Sciences, with an ethical number assigned as ZZU-LAC20220311(14).

### 2.11 In vivo imaging and biodistribution analysis

When the tumor's volume reached 100 mm^3^, the nude mice were randomly allocated into two groups: IR783 group and RIMNPs group, with each group receiving a tail vein injection of 100 μL free IR783 or RIMNPs (IR783, 1.0 mg kg^−1^) via the tail vein. The fluorescence signals emitted by IR783 at the tumor site were captured at 2, 4, 6, 8, 10, 24, 36, and 48 h post-injection using an ex/in vivo imaging system (PerkinElmer PE IVIS SPECTRUM, United States). At the end of a period of 48 h post-injection, euthanasia was performed on mice and major organs (heart, liver spleen lung kidneys), as well as the tumor itself was collected for semi-quantitative biodistribution analysis and imaging using an ex/in vivo imaging system.

### 2.12 In vivo photothermal studies

BALB/cA-nu mice bearing HepG2 tumors (100 mm^3^) were intravenously administered with RIMNPs (80 μg/mL, 100 mL), while the control group was treated with 100 μL PBS. After 6 h, the tumors were exposed to laser irradiation (808 nm, 0.5 W/cm^2^) for a duration of 5 min. Infrared thermographic maps and regions displaying maximum temperatures were obtained using an infrared thermal imager.

### 2.13 In vivo anti-tumor effect and biosafety of RIMNPs

When the tumor reached a volume of approximately 50 mm^3^, six nude mice per group were randomly assigned to the following five groups: (I) PBS, (II) Reg, (III) RINPs, (IV) RIMNPs, and (V) RIMNPs + Laser. Each group received an intravenous injection of 100 μL (including Reg at a concentration of 80 μg/mL) every other day for a duration of two weeks. After a 6-h injection, the tumors in the RIMNPs Laser group were exposed to laser irradiation (808 nm, 0.5 W/cm^2^) for a duration of 5 min. Apart from this aspect, all other experimental conditions remained consistent with those applied to the remaining groups. The body weight and tumor volumes of mice were recorded on alternate days. The mice underwent small animal magnetic resonance imaging (MRI) examination and were euthanized on the 14th day, followed by excision and quantification of the tumor tissue. The obtained tumor tissue was fixed in formalin solution, embedded in paraffin, and subjected to histological staining with H&E for assessing tumor necrosis, Ki67 for evaluating proliferation, and TUNEL for detecting apoptosis. The polarization types of tumor-associated macrophages (TAMs) in tumor tissues were assessed through immunofluorescence staining for F4/80, CD80, and CD206. Neovascularization and vascular structure in the tumor tissues were evaluated by detecting CD31 and ɑ-SMA using immunofluorescence techniques.

Additionally, following the euthanasia of each cohort of nude mice, the heart, liver, spleen, lung, and kidney were excised for histological examination using H&E staining to assess significant organ damage. Blood samples were collected for biochemical analysis, including blood routine tests to determine the levels of white blood cells (WBC), red blood cells (RBC), and platelets (PLT); hepatic function tests to measure alanine aminotransferase (ALT), aspartate aminotransferase (AST), direct bilirubin (DBIL), and total bilirubin (TBIL); and renal function tests to assess uric acid (UA), urea (UREA), and creatinine levels.

### 2.14 Hemolysis experiments

Orbital blood was collected from healthy BALB/c mice and subsequently centrifuged at 3500 rpm for 5 min to obtain plasma. The upper layer containing plasma, platelets, and white blood cells was discarded. The precipitate obtained in the aforementioned steps was washed twice with PBS and then diluted with PBS to obtain a 2 % (v/v) suspension of erythrocytes. Subsequently, the red blood cell suspension was mixed with 0.2 mL of PBS (as a negative control), 0.2 mL of Triton X-100 (as a positive control), 0.2 mL of RINPs at various concentrations (5, 10, 20, 40, and 80 μg/mL), and different concentrations (5, 10, 20, 40, and 80 μg/mL) of RIMNPs. The mixture was incubated at 37 °C for 4 h, followed by collection of the supernatant through centrifugation at 1000*g* for 5 min. The condition of the Eppendorf tubes was visually documented and recorded. The supernatants were subsequently transferred to a 96-well plate, and the optical density (OD) value at 541 nm was measured using a microplate analyzer. Finally, the hemolysis rate was then calculated using the following formula: Hemolysis rate (%) = (A_preparation_ - A_negative_)/(A_Positive_ - A_negative_) × 100.

### 2.15 Statistical analysis

All experiments were independently conducted at least three times (n ≥ 3). The data were presented as the mean ± standard deviation (SD). The statistical significance of differences was determined by using GraphPad Prism 8.0 software through the application of one-way ANOVA-LSD and Independent Samples *t*-test. Statistically significant differences were denoted by P < 0.05: ∗; P < 0.01: ∗∗; and P < 0.001: ∗∗∗.

## 3 Results and discussion

### 3.1 Synthesis and characterization of RINPs

**Fig. 1:**
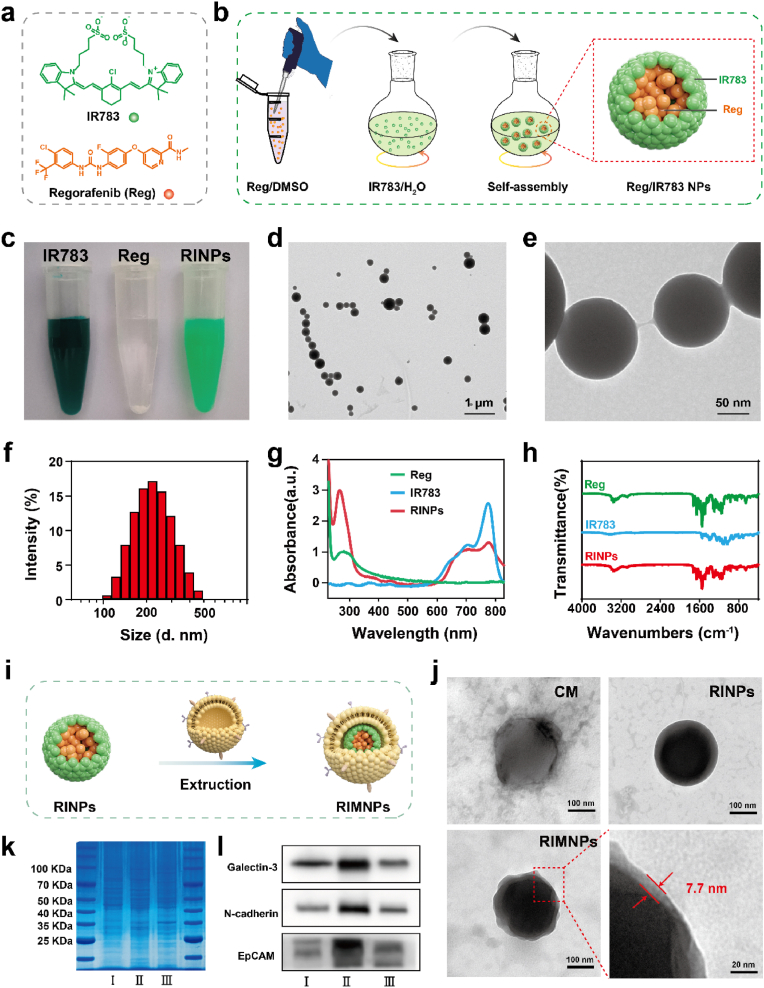
Synthesis and characterization of the RIMNPs. (a) The chemical structure of IR783 and regorafenib. (b) The self-assembly process of RINPs by IR783 and regorafenib. (c) The digital images of IR783, regorafenib, and RINPs dispersion in aqueous solution. (d, e) Representative TEM images with different magnifications. (f) Size distribution of the RINPs suspended in aqueous solution. (g) UV–Vis spectrum of IR783, regorafenib and RINPs. (h) Fourier Transform Infrared Spectrometer (FTIR) of Reg, IR783, and RINPs. (i) Schematic preparation of RIMNPs. (j) Representative TEM images of CMNPs, RINPs, and RIMNPs. (k) SDS-PAGE protein analysis of the whole cell, CM, and RIMNPs. (l)Western blotting analysis for Galectin-3, N-cadherin, and EpCAM in the whole cell, CM, and RIMNPs.

The anti-solvent precipitation method is widely recognized as a straightforward and efficient technique for producing carrier-free nanodrugs [42,43]. Regorafenib is a clinical second-line anticancer drug, characterized by a π-electronic planar structure, tending to pack together in one direction and forming one-dimensional nanoarchitecture through π-π stacking and hydrophobic interactions (Fig. 1a). However, these drug aggregates often exhibited irregular shapes and non-uniform sizes and were prone to aggregation and precipitation in aqueous dispersions (Fig. S1). To enhance stability and control structure, indocyanine, particularly IR783, was introduced as a cosolvent. IR783, a relatively polar and amphipathic molecule, acts similarly to a surfactant (Fig. 1a). It consists of a symmetric benzoindole hydrophobic group and two sulfonic acid groups on the sides, facilitating the co-assembly of stable colloidal nanodrugs with hydrophobic molecular through multiple supramolecular interactions, including hydrogen bonds and π-π stacking interactions [44].

The carrier-free co-assembly nanodrug (Reg/IR783) was successfully synthesized using the classical nanoprecipitation method [45]. As illustrated in Fig. 1b, a solution of Reg in DMSO was gradually added to an IR783 solution in water under magnetic stirring. Following centrifugation to remove the excessive IR783, the resulting pellets were resuspended in water. By adjusting the dosage ratio between IR783 and Reg prodrug, it was found that when the solution concentration of IR783 was 400 μg/mL, the obtained NPs had the smallest diameter and the lowest polydisperse index (PDI), which was used for follow-up experiments (Fig. S2). Visibly, the co-assembled nanoaggregates displayed excellent colloidal stability in aqueous solutions compared to precipitated drugs, demonstrating that this nano-assembly strategy effectively enhances the solubility of regorafenib (Fig. 1c). The morphological characteristics of the IR783@Reg were then analyzed. Transmission electron microscopy (TEM) revealed that Reg@IR783 possessed a regular spherical morphology and high dispersibility (Fig. 1d and e). Dynamic light scattering (DLS) measurements indicated that the prepared RINPs had a narrow size distribution ranging from 150 to 450 nm with an average diameter of approximately 167 nm, maintaining stable stability in water with no significant size change over five days (Fig. 1f&S3). The Reg/IR783 NPs were negatively charged at −34.75 mV, which attributed to the negatively charged sulfonate groups of IR783 (Fig. S4). Impressively, the co-assembled nanodrugs achieved unusually high drug loading of Reg, reaching 99.8 %. As shown in Table S1, we found that both the loading capacity and encapsulation efficiency of Reg were remarkably high, reaching 99.67 % and 85.24 %, respectively, which demonstrated that Reg was the main component that assembled into Reg/IR783 NPs in the presence of small amount of IR783. Additionally, the UV–vis spectrum of the RINPs displayed the characteristic peaks of IR783 and regorafenib at 280 nm and 780 nm respectively, confirming their successful integration (Fig. 1g). The co-assembly of IR783 with regorafenib also resulted in a significantly broader and red-shifted Soret band of IR783, suggesting interactions between the aromatic rings of regorafenib and IR783 through π-π stacking [46]. Further, Fourier transform infrared (FT-IR) spectra (Fig. 1h) showed that the absorption band at 1599 cm^−1^ shifted to 1602 cm^−1^ following the co-self-assembly of Reg with IR783, and the N-H absorption band from Reg shifted from 3378 to 3348 cm^−1^. These shifts indicate that hydrogen bonds between the amine/carboxyl groups of Reg and the sulfonate groups of IR783 contribute to their co-self-assembly. However, the absorption peak of IR783 was less observable in the RINPs, which correlates with the previously measured content of IR783.

To mitigate clearance by the reticuloendothelial system and enhance the tumor-targeting capabilities of RINPs during systemic circulation, the cell membrane from liver cancer cell HepG2 was isolated and applied to the surface of RINPs through physical extrusion [41] (Fig. 1i). This was followed by an analysis of the physicochemical properties of RINPs before and after cell membrane coating. As indicated in Fig. 1j, both RINPs and pure CMNPs presented a uniform and regular spherical structure with a size of approximately 210 nm. However, RIMNPs exhibited a distinct core-shell structure with a diameter of about 220 nm and a consistent outer shell thickness of around 10 nm, confirming the presence of the cancer cell membrane. Dynamic light scattering (DLS) data indicated an increase in diameter from 240 nm for RINPs to 245 nm for RIMNPs (Fig. S5), while the polydispersity index (PDI) values were recorded at 0.41 for RINPs and 0.09 for RIMNPs, reflecting improved uniformity post-coating. Additionally, the zeta potential of the RIMNPs was measured at −27.89 mV, closely resembling that of cancer cell membrane-derived vehicles (−16.5 mV) but significantly different from the RINPs (Fig. S6). The successful coating of the cell membrane was further validated through protein analysis, showing that the protein composition of RIMNPs closely matches that of the original hepG2 cell membrane that indicates that it retains the proteins of the cell membrane (Fig. 1k).

Western blot analysis was conducted to confirm the presence of cell adhesion molecules (Galectin-3, EpCAM, and N-cadherin) expressed by HepG2 tumor cells, essential for homotypic targeting and reducing macrophage uptake, thereby facilitating the specific recognition and binding of CM-coated nanoparticles to cancer cells [47] (Fig. 1l). To evaluate the stability and biocompatibility of the self-assembled RIMNPs, their size and zeta potential were monitored at different time points. As shown in Fig. S7, there were no significant changes in size when RIMNPs were stored in water, and DMEM for three days, suggesting enhanced stability of the self-assembled nanoparticles.

### 3.2 NIR-driven structural transformation and photothermal from RIMNPs

**Fig. 2:**
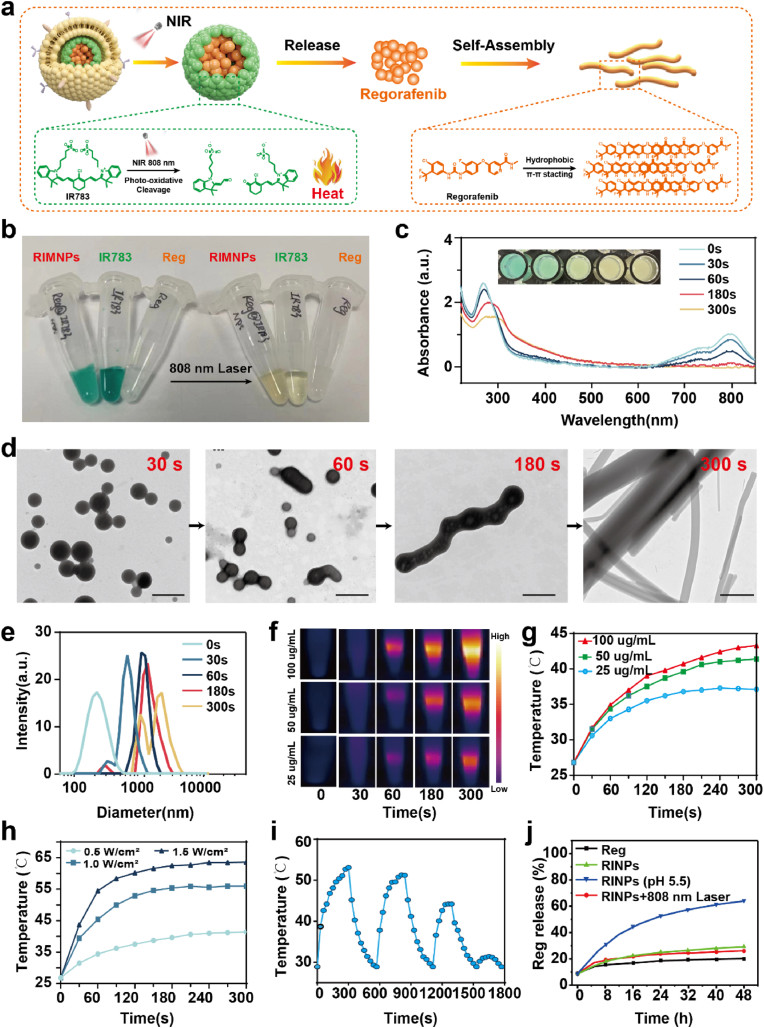
NIR triggers the structural and photothermal effects of RIMNPs. (a) Schematic diagram of photodegradation heat production of RIMNPs under near-infrared irradiation and the reassembly of Reg. (b) The images of IR783, regorafenib, and RIMNPs dispersion in deionized water. (c) UV–Vis absorption spectra and color change of RIMNPs irradiation for different times. (d) TEM images of RIMNPs before and after 808 nm laser irradiation at 1.0 W/cm^2^ for 5 min (scale bar = 200 nm). (e) Hydrodynamic size changes of RIMNPs laser irradiation. (f) Photothermal images and (g) dose-dependent temperature increase profiles of RIMNPs irradiated with NIR (808 nm, 0.5 W/cm^2^). (h) Energy density-dependent temperature increases profiles of RIMNPs (50 μg/mL) irradiated with 808 nm laser. (i) On-off curves of RIMNPs (100 μg/mL) under 808 nm laser irradiation at 1.0 W/cm^2^ for 4 cycles. (j) Drug release curves of Reg under different conditions.

Previous research indicates that core-shell nanoaggregates, such as those made from IR783/sorafenib, typically feature IR783 as the shell with sorafenib encapsulated within the solid core [38]. Given the structural similarities with sorafenib, the regorafenib/IR783 NPs may also maintain a similar structure with densely packed regorafenib at the core and a minor quantity of IR783 forming the shell. In this arrangement, IR783 acts to restrain the self-assembly of regorafenib, creating spherical nanoaggregates and enhancing water solubility via its external hydrophilic groups. However, when the 808 nm laser was applied to RIMNPs, the surface IR783 was able to be degraded by photobleaching. With the absence of the protective layer of IR783, regorafenib can further grow and form nanofibers through local high concentration self-assembly, thus achieving long-lasting retention and controlled release of chemotherapy drugs (Fig. 2a).

To confirm the relevant performances, the nanomedicine was irradiated by an infrared laser (808 nm, 1.0 W cm^−2^). Intriguingly, we unexpectedly observed a gradual color change in RINPs from dark green to yellow under NIR irradiation (Fig. 2b). Optical microscope and TEM observations revealed significant morphological changes in RINPs after NIR light exposure, transitioning to uniform rod or fibrous structures (Figs. S8 and S9). To explore this phenomenon, we analyzed the photo-responsive properties of RINPs using UV–Vis spectra. Initially, the absorption spectra of RINPs without light exposure (Fig. 2c) displayed distinct peaks at approximately 280 and 780 nm, corresponding to the absorption maxima of Reg and IR783, respectively. However, upon irradiation with an 808 nm laser at 1.0 W/cm^2^, the 780 nm absorption peak rapidly diminished within 30 s, and the 280 nm peak shifted to approximately 290 nm. With the extension of time to 300s, the solution changed from blue to yellow, accompanied by the absorption of IR783 disappearing. These changes suggest both the degradation of IR783 and potential structural modifications in RIMNPs due to NIR irradiation. Further morphological and size analyses of RINPs were conducted using transmission electron microscopy (TEM) and dynamic light scattering (DLS). It was observed that the initially spherical RIMNPs, with an average diameter of about 220 nm, transformed into micron-sized cylindrical structures within 5 min of NIR exposure. These structures exhibited a base diameter ranging from 500 to 800 nm and a length varying from 5 to 20 μm (Fig. 2d). Correspondingly, the hydrodynamic diameter of the RINPs also increased significantly, from approximately 210 nm to about 1.4 μm (Fig. 2e). To explore the light-induced deformation mechanism of RINPs, the ^1^H NMR spectra were analyzed before and after NIR irradiation. Figs. S10 and S11 reveal that regorafenib predominantly constituted the nanoaggregate, while IR783 was present in a much smaller proportion, aligning with earlier drug content assessments. Post-exposure to NIR light, IR783 content significantly decreased, with virtually no response peak evident, suggesting its degradation under light. This finding was also confirmed by the high-resolution MS analyses (Figs. S12 and S13). The ionic peak of 501.0947 and 726.2559 (corresponding to Reg and IR 783 respectively) was also observed in the high-resolution MS spectrum. Moreover,

Our RIMNPs are expected to act as a good photosensitizer for PTT, which is promising for efficient cancer treatment. To assess the photothermal properties of RIMNPs, an infrared thermal imaging system was employed to monitor real-time temperature changes during laser irradiation (808 nm, 1.0 W cm^−2^). Fig. 2f&g illustrate that the temperatures of RIMNP solutions increase with both concentration and duration of laser exposure. Specifically, after 10 min of exposure to a 1.0 W/cm^2^ laser, the temperatures of the RIMNP solutions rose sharply, reaching 23.1, 49.6, and 59.3 °C at varying concentrations. In contrast, an equivalent volume of pure water under the same conditions experienced only a modest increase of 1.8 °C. Furthermore, the temperature dynamics of the RIMNP solutions were also shown to depend on laser power, as evidenced by Fig. 2h&S14, which demonstrated that peak temperatures increased with power densities. Additionally, the photothermal heating/cooling cycles' stability was tested by subjecting RINPs (100 μg/ml) to three cycles of 808 nm laser irradiation at a power density of 1.0 W/cm^2^. Despite the gradual degradation of IR783, RIMNPs demonstrated remarkable stability, maintaining a maximum temperature of 53.0 °C after 5 min during the initial cycle. In the second cycle, the highest recorded temperature was 51.5 °C (Fig. 2i), consistent with previous research and further validated by this study. These results confirm the exceptional photothermal properties of RIMNPs, highlighting their potential as photothermal agents in photothermal therapy (PTT). Meanwhile, the release behavior of Reg from RINPs under various treatment conditions was investigated over time (Fig. 2j). The results demonstrated that the release drug increased with a low pH value. Notably, the release of Reg from RINPs in the presence of laser irradiation was not significantly higher compared to RINPs without light exposure and pure Reg. This phenomenon may be caused by the reoccurrence of self-assembly of regorafenib. Furthermore, in order to eliminate the secondary assembly of nanoparticles caused by heat, we studied the morphological changes of RINPs and RIMNPs at 25 °C and 65 °C. As can be seen from Fig. S15, after heating for 10 min, neither the color nor the morphology of RINPs and RIMNPs changed. This indicates that temperature is not a factor for the secondary deformation of RINPs.

### 3.3 In vitro targeting of RIMNPs to homotypic cells

**Fig. 3:**
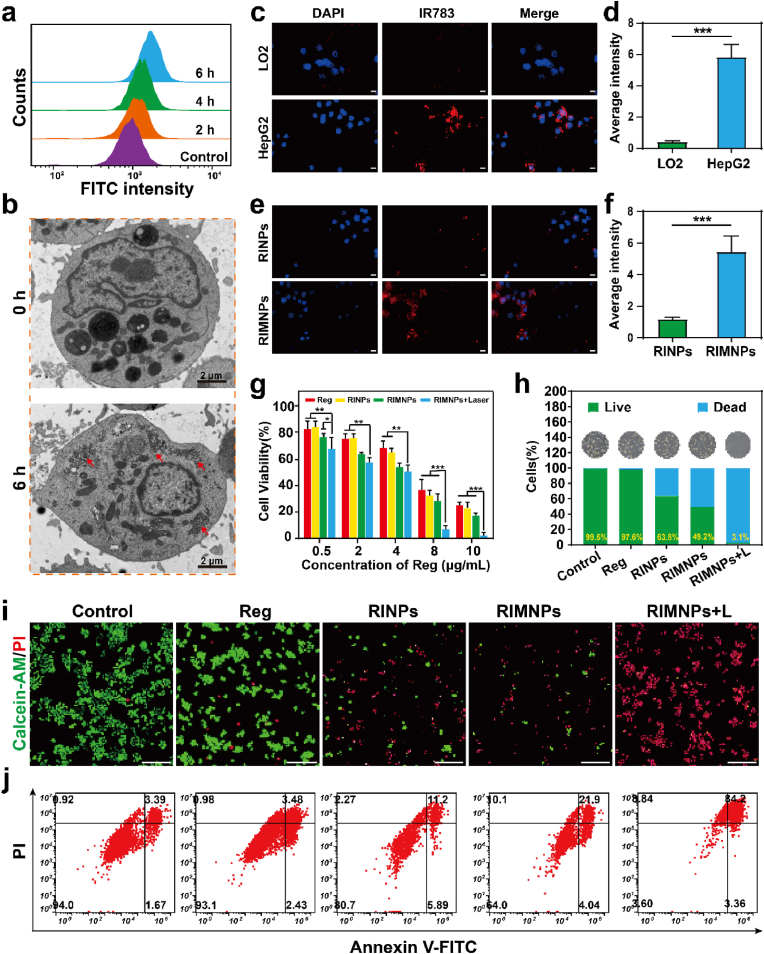
The anti-tumor activity of RIMNPs i*n vitro*. (a) Flow cytometry analyses of HepG2 cells incubated with RIMNPs for 2, 4, and 6 h. (b) Bio-TEM images of HepG2 cells incubated with RIMNPs for 6 h. (c) Cellular uptake of RIMNPs in LO2 and HepG2 cells after 6 h of incubation (scale bar = 50 μm) and (d) Related fluorescence semi-quantitative analysis. (∗∗∗, p < 0.001). (e) Fluorescence images and (f) the relevant semi-quantitative analysis of RINPs and RIMNPs after incubation with HepG2 cells for 6 h (scale bar = 50 μm; ∗∗∗, p < 0.001). (g) Cell viability of HepG2 cells after being treated with Reg, RINPs, RIMNPs, and RIMNPs + Laser for 24 h with various concentrations of Reg (0.5, 2, 4, 8, 10 μg/mL). (∗, p < 0.05, ∗∗, p < 0.01; ∗∗∗, p < 0.001). (h) Quantification bar plots for live/dead staining and the inserted microscope images. The values are presented by mean value ± SD. (i) Fluorescence images of HepG2 cells stained with Calcein-AM and PI with different treatments (Scale bar = 20 μm). (j) Flow cytometric analysis of HepG2 cell apoptosis after treatment with PBS, Reg, RINPs, RIMNPs, and RIMNPs + Laser for 6 h.

The effective internalization of therapeutic nanoparticles into tumor cells is crucial for a robust therapeutic outcome. From the data shown in Fig. 3a & S16, it is clear that HepG2 cells rapidly internalize RIMNPs, with fluorescence intensity within the cells increasing over a period of 4 h. Further, after a 4-h incubation of RIMNPs with HepG2 cells, bio-TEM analysis revealed a considerable presence of nanoparticles in various stages of degradation within the endosomes (Fig. 3b), confirming successful cellular uptake of RIMNPs. To assess the specificity of RIMNPs towards homologous HepG2 cells, their targeting capability was compared against LO2 cells using confocal laser scanning microscopy (CLSM). The results indicated that the HepG2 group exhibited higher uptake efficiency and fluorescence intensity, demonstrating the specific binding affinity of RIMNPs to homologous HepG2 cells (Fig. 3c&d). Additional experiments co-culturing both RINPs and RIMNPs with HepG2 cells (Fig. 3e&f) utilized laser confocal microscopy to observe that nanoparticles encapsulated within cell membranes were preferentially internalized by HepG2 cells, highlighting their inherent targeting capabilities. A significant advantage of biomimetic nanodrugs, particularly those encapsulated with tumor cell membranes, is their ability to evade macrophage clearance, thus enhancing accumulation in tumor tissues. In experiments involving macrophage-like RAW 264.7 cells incubated with both RIMNPs and RINPs, CLSM observations (Fig. S17) showed noticeably reduced fluorescence intensity in cells cultured with RIMNPs compared to those with RINPs. This supports the notion that coating with a cancer cell membrane significantly reduces immune clearance, thereby potentially increasing the efficacy and persistence of the nanodrugs in tumor targeting.

### 3.4 In vitro anti-tumor activity of RIMNPs

The combination of chemo-phototherapy has demonstrated significant potential in cancer treatment, particularly in overcoming the chemoresistance of chemotherapeutics. To assess the chemo-photothermal therapeutic efficacy of RIMNPs in vitro, HepG2 cells were treated with or without laser irradiation (5 min, 1.0 W/cm^2^) in the presence of RIMNPs. Cell viability was measured using the CCK-8 assay. As illustrated in Fig. 3g, concentration-dependent cytotoxicity was observed in HepG2 cells treated with varying concentrations of free Reg, RINPs, RIMNPs, and RIMNPs + Laser. Notably, the cytotoxicity of RIMNPs, both with and without laser irradiation, at a low concentration of 2 μg/mL, was more pronounced compared to the same amount of Reg and RINPs. Specifically, RIMNPs with PTT induced approximately 93 % cell death at 8 μg/mL, which is significantly higher than that observed with Reg (58 %), RINPs (62 %), and RIMNPs alone (68 %). Consequently, a concentration of 8 μg/mL was chosen for subsequent experiments. In contrast, we found that RIMNPs + Laser exhibited very low toxicity to LO2 cells even at high concentrations, with cell viability higher than 70 % at 10 μg mL^−1^ (Fig. S18). Fluorescent staining of live/dead cells further demonstrated the superior therapeutic efficacy against tumor cells, with a remarkable 96.9 % cell death for RIMNPs with PTT, compared to only 2.4 % for Reg, 36.2 % for RINPs, and 50.8 % for RIMNPs alone (Fig. 3h–i&S19). Flow cytometry analysis corroborated these findings, showing that the apoptosis rate induced by RIMNPs under NIR irradiation was 84.2 %, while the rates for the free Reg, RINPs, and RIMNPs groups were 3.48 %, 11.2 %, and 21.9 %, respectively (Fig. 3j). Additionally, Bio-TEM analysis after incubation of RIMNPs with HepG2 cells for 4 and 24 h revealed significant nuclear enlargement and membrane rupture post-NIR irradiation, with more pronounced cell fragmentation and inflammation-induced cell death observed after 24 h of co-cultivation (Fig. S20). Overall, these results confirm that the homologous targeting of RIMNPs not only significantly enhances their endocytosis by tumor cells but also amplifies the synergistic effects of photothermal and chemotherapy against cancer cells.

### 3.5 In vivo imaging and biodistribution of RIMNPs

**Fig. 4:**
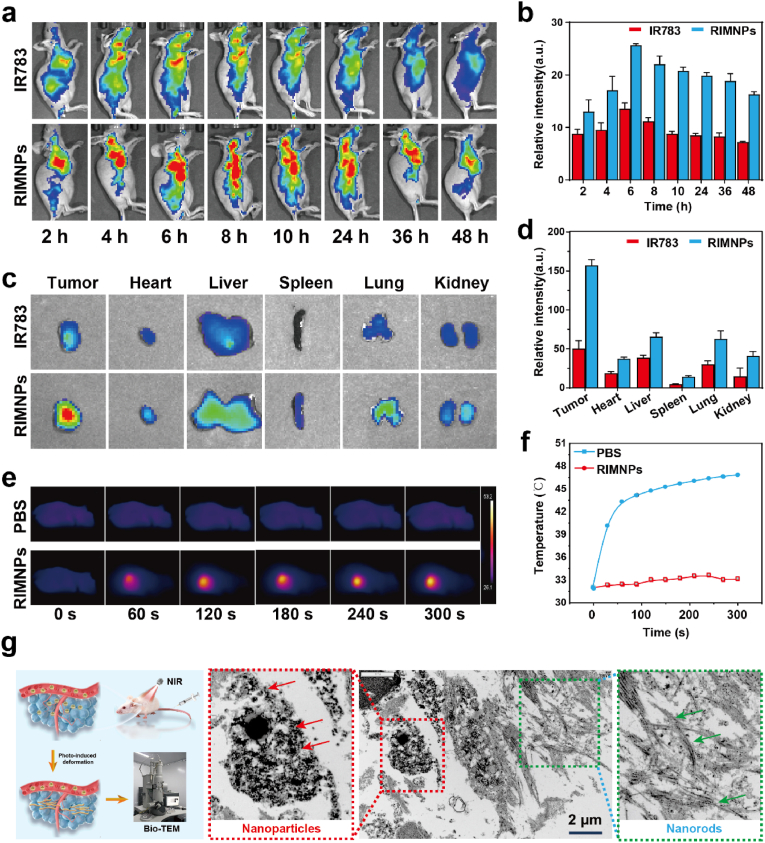
Biodistribution and in vivo photothermal performance of RIMNPs. (a) NIR fluorescence imaging of nude mice bearing HepG2 tumors intravenously administrated with RIMNPs and free IR783 at 2, 4, 6, 8, 10, 24, 36, and 48 h post-injection. (b) Average fluorescence intensity from IR783 in the tumors over time, measured by an in vivo imaging system (n = 3). (c) Ex vivo NIR fluorescence images of IR783 in the harvested organs (heart, spleen, liver, kidney, and lung) and tumors at 48 h post-administration (n = 3). (d) Quantitative analysis of fluorescence intensity of IR783 in the harvested organs and tumors at 48 h post-administration (n = 3). (e) Representative infrared thermal images and (f) temperature change at the tumor sites of mice bearing HepG2 tumors treated with i.v. injection of RIMNPs followed by 808 nm laser irradiation (1.0 W/cm^2^, 5 min) at 6 h post-administration. (h) Schematic of light driven second-assembly of RIMNPs. (g) Bio-TEM images of HepG2 tumors intravenously administrated with RIMNPs with laser irradiation.

Given the effective homologous targeting and strong anti-proliferative activity of the nanodrugs against liver cancer cells, we explored their potential for in vivo application. Initially, we assessed the hemocompatibility of our biomimetic nanodrug using a hemolysis assay. As indicated in Fig. S21, the hemolysis experiments confirmed that RIMNPs maintain good biocompatibility at concentrations up to 500 μg/mL. Encouraged by the favorable hemocompatibility of RIMNPs, we proceeded with in vivo applications. IR783 and RIMNPs were administered intravenously into HepG2 tumor-bearing nude mice. Fluorescence imaging was used to monitor the fluorescence signals in the mice at various time intervals. As shown in Fig. 4a&b, both free IR783 and RIMNPs demonstrated tumor-targeting effects, with RIMNPs exhibiting significantly higher fluorescence intensity. However, free IR783 was rapidly cleared from the body, with no discernible signals detected after 8 h. In contrast, RIMNPs achieved enhanced tumor accumulation through both passive targeting via the Enhanced Permeability and Retention (EPR) effect and active homologous targeting, reaching peak accumulation at 6 h post-injection and maintaining detectable levels up to 48 h [48]. Subsequently, 48 h after injection, the tumors and major organs of the mice were harvested to investigate the distribution of free IR783 and RIMNPs. The fluorescence intensity was predominantly localized at the tumor site, with partial distribution observed in the liver and lungs (Fig. 4c&d). Notably, the fluorescence intensity of RIMNPs at the tumor site was 3.25 times higher than that of free IR783, underscoring its exceptional tumor-targeting capability. These findings strongly suggest that RIMNPs not only excel in cellular-level targeting but also demonstrate selective accumulation in tumor tissues, highlighting their potential for real-time cancer imaging and photothermal therapy (PTT) applications in vivo.

### 3.6 In vivo photothermal performance and second assembly of RIMNPs

Encouraged by the outstanding tumor accumulation and activation performance of our biomimetic nanodrug, we conducted photothermal imaging to evaluate the photothermal properties of RIMNPs in tumor tissues under laser irradiation (808 nm, 1.0 W/cm^2^). To assess the in vivo photothermal efficacy, we intravenously injected 100 μL of PBS or RIMNPs (80 μg/mL) into tumor xenograft mice when the tumors reached approximately 100 mm^3^. Six hours post-injection, the temperature changes in the tumor region were monitored during 5-min laser irradiation at 0.5 W/cm^2^. As shown in Fig. 4e&f, the temperature in the tumor area quickly escalated to over 43 °C within the first 30 s of NIR irradiation and stabilized at around 48 °C throughout the irradiation period. These results demonstrate that RIMNPs not only achieve precise tumor accumulation and enable fluorescence imaging but also effectively facilitate photothermal therapy (PTT) in vivo through specific homologous targeting combined with the Enhanced Permeability and Retention (EPR) effect. Additionally, biological electron microscopy was employed to observe the secondary assembly of nanoparticles under light irradiation. As depicted in Fig. 4g&S22, rod-shaped nanomaterials were observed within the tissue, confirming that the nanomaterials can undergo secondary assembly upon light irradiation, aligning with our initial hypothesis and in vitro findings. This indicates that RIMNPs can rapidly generate heat under NIR light, triggering the secondary assembly of regorafenib and enhancing the long-term retention of the drug at the tumor site, thereby optimizing the therapeutic outcome.

### 3.7 In vivo synergistic antitumor efficiency of RIMNPs

**Fig. 5:**
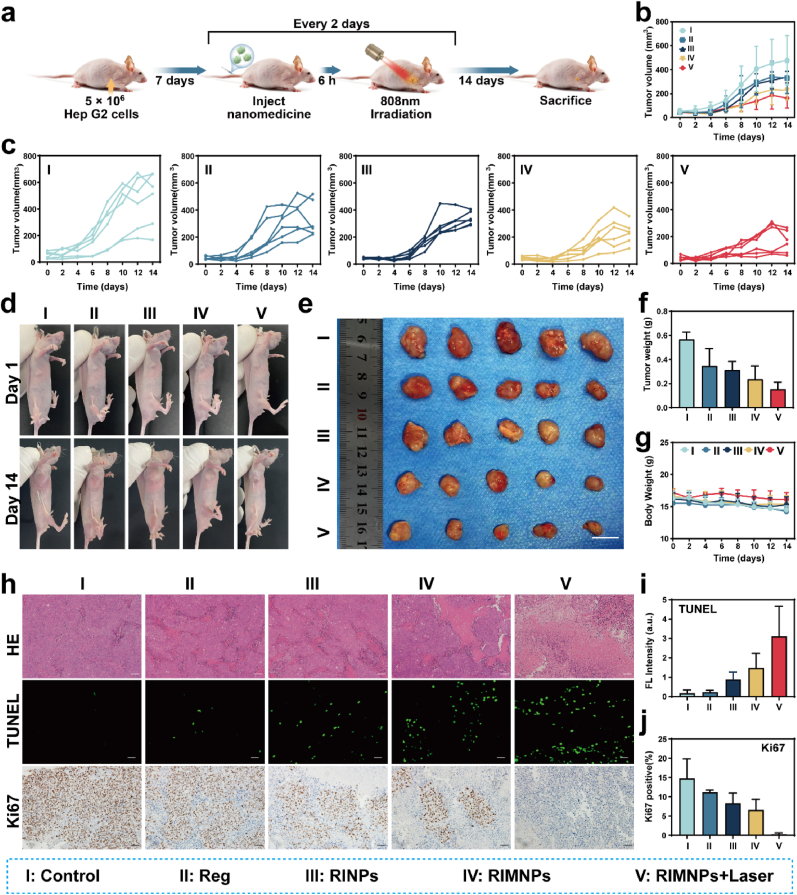
*In vivo* synergistic photothermal-chemotherapy efficacy of RIMNPs to nude mice bearing HepG2 tumors. (a) Schematic experimental design for evaluating the in vivo anti-tumor effect of RIMNPs. (b, c) Tumor growth curves of tumor-bearing mice treated with PBS, Reg, RINPs, RIMNPs, and RIMNPs + Laser. (d) The images of tumor model mice on the first and 14th day after different treatments. (e) The images and (f) weights of harvested tumors in different groups after treatments. (g) Body weight of tumor-bearing mice treated with PBS, Reg, RINPs, RIMNPs, and RIMNPs + Laser. (h) H&E, Ki67, and TUNEL staining images of the excised tumors from mice treated with various drug formulations on day 14 (Scale bar = 50 μm). Quantitative analyses of (i) The relative intensity of TUNEL immunofluorescence and (j) Ki67-positive cells. All the data are shown as mean ± SD.

Motivated by the promising in vitro antitumor effects of RIMNPs, we further investigated their therapeutic efficacy in combining photothermal therapy (PTT) and chemotherapy on HepG2 tumor bearing nude mice. Upon the primary tumor volume reaching approximately 50 mm^3^, mice were randomly grouped (n = 6) and divided into five groups: PBS, Reg, RINPs, RIMNPs, and RIMNPs + Laser. The treatment schedule was carefully planned and is illustrated in Fig. 5a. Over the 14-day intervention period, tumor volume fluctuations were meticulously monitored (Fig. 5b). Notably, Fig. 5c–f highlighted the treatment outcomes across the groups. The RIMNPs + Laser group demonstrated significant tumor growth inhibition compared to the PBS and other treatment groups. It is important to note that the Reg group did not show substantial tumor growth inhibition, primarily due to an insufficient dosage. The antitumor effects were more pronounced upon examination of the excised tumors after the 14-day treatment period (Fig. 5e&S23), which was further confirmed by MRI (Fig. S24). The tumors were also weighed post-study (Fig. 5f), with the RINPs, RIMNPs, and RIMNPs + Laser groups showing substantial reductions in tumor weight by 46.1 %, 58.3 %, and 73.9 %, respectively. These results underscored the enhanced tumor-targeting aggregation and superior anti-cancer efficacy of these treatments. Notably, all mouse groups maintained normal body weights throughout the study, indicating the low toxicity of RIMNPs (Fig. 5g). To delve deeper into the synergistic effects of PTT and chemotherapy at the tissue level, pathological examinations including H&E, Ki67, and TUNEL staining were conducted. TUNEL and H&E images revealed no significant change in cell morphology in the control group, whereas notable morphological changes such as cell contraction, chromatin condensation, nucleus disintegration, and enlarged necrotic tissue areas were observed in the RINPs, RIMNPs, and RIMNPs + Laser groups (Fig. 5h and i). Moreover, Ki67 staining indicated that the RIMNPs + Laser treatment exhibited the strongest inhibitory effect on the proliferation of HepG2 cells compared to the other groups (Fig. 5j). Clearly, the encapsulation of homologous cell membranes significantly enhances drug metabolism and blood circulation time of carrier-free nanomedicines. Furthermore, the long-term tumor inhibition by RIMNPs + Laser therapy can be attributed not only to the local hyperthermia produced by PTT but also to the secondary assembly of light-driven chemotherapy drugs. This enhances drug enrichment and retention at the tumor site, boosting their anti-proliferative and pro-apoptotic effects. Overall, these findings demonstrate that RIMNPs can serve as an innovative biomimetic nanodrug for precise delivery and synergistic photothermal/chemotherapy against hepatocellular carcinoma (HCC), showcasing their potential as a powerful treatment modality.

### 3.8 Enhanced cancer immunotherapy of RIMNPs by reprogramming TAMs

**Fig. 6:**
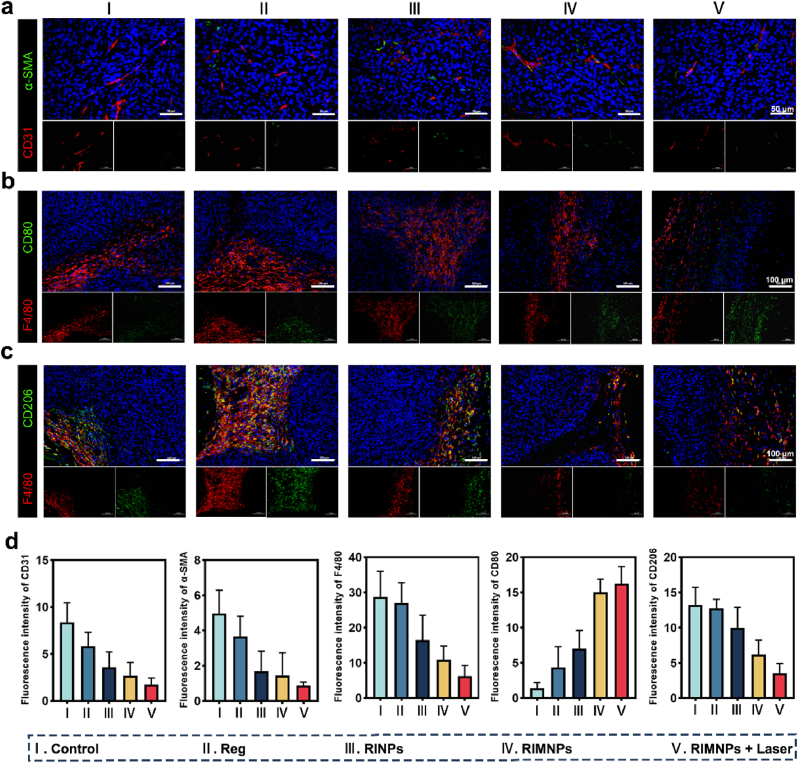
RIMNPs combine photothermal therapy to enhance cancer immunotherapy through vascular normalization and reprogramming macrophage. (a) Representative images of CD31 (red) and ɑ-SMA (green) by immunofluorescence staining of endothelial cells and pericytes, respectively. Scale bar = 50 μm. (b) Representative immunofluorescence images of M1 (F4/80^+^CD80^+^) and (c) M2 (F4/80^+^CD206^+^) macrophages in tumors of mice with various treatments. Scale bar = 100 μm. (d) Quantification analysis of CD31-positive endothelial cells, ɑ-SMA-positive pericytes, F4/80^+^ TAMs, M1, and M2 macrophage in tumors of mice with various treatments.

Tumor-associated macrophages (TAMs) are crucial components of the tumor microenvironment, influencing angiogenesis and resistance to chemotherapeutic agents [49]. Studies have indicated that Regorafenib (Reg) not only inhibits tumor growth by normalizing tumor vasculature but also enhances anti-tumor effects by reducing TAM infiltration and reprogramming macrophage polarization towards the M1 phenotype [50,51]. This multifaceted approach provides a deeper understanding of the mechanisms underlying photothermal combined with chemotherapy for tumor treatment. Through immunofluorescence analysis, we observed significant inhibition of tumor angiogenesis in the RINPs, RIMNPs, and RIMNPs + Laser groups. There was a notable reduction in CD31-positive endothelial cells and α-SMA-positive pericytes. Moreover, vascular structures double-positive for CD31 and α-SMA were more prevalent in these treatment groups (Fig. 6a and d), demonstrating that nanomedicines enhance the aggregation of Reg in tumor tissues, thus exerting potent anti-angiogenic effects and promoting the normalization of tumor vascular structures, aligning with prior research findings. Considering the notable immunomodulatory properties of Reg, we hypothesized that RIMNPs could boost anti-tumor immunity by attenuating TAM infiltration and reprogramming TAMs within the tumor microenvironment (TME). To test this hypothesis, we analyzed the expression of F4/80, CD206, and CD80 in tumors using immunofluorescence. The results indicated that, unlike the control and free Reg groups, all nanomedicine treatment groups exhibited significantly fewer F4/80-positive cells in tumor tissues (Fig. 6b and d). This suggests that nanomedicine enhances Reg's chemotherapeutic effect by reducing TAM recruitment within tumor tissues, corroborating our previous findings regarding antiproliferative and proapoptotic effects. Furthermore, there was a significant reduction in the proportion of M2 macrophages, and an increase in M1 macrophages in all nanomedicine treatment groups, as evidenced by the quantitative analysis of CD80 and CD206-positive cells (Fig. 6c and d). These findings provide compelling evidence that Reg-containing nanomedicines can effectively induce macrophage polarization from an M2 phenotype to an M1 phenotype.

Notably, the combined treatment of RIMNPs with PTT resulted in the least infiltration of F4/80-positive cells and the lowest proportion of M2 macrophages. This outcome can be attributed to the synergistic effect of PTT and Reg through a mutually reinforcing mechanism. While local hyperthermia induced by PTT effectively triggers cancer cell death, it also initiates an inflammatory response at the tumor site, potentially increasing the risk of tumor metastasis and recurrence [52,53]. However, Reg counters this limitation by attenuating TAM infiltration and promoting macrophage polarization towards the M1 phenotype to bolster anti-tumor immunity. In summary, biomimetic RIMNPs significantly improve the precise delivery of Reg to tumors, thereby enhancing its effectiveness in inhibiting tumor angiogenesis and reprogramming TAMs, ultimately leading to synergistic tumor therapy with PTT.

### 3.9 In vivo biosafety evaluation of RIMNPs

**Fig. 7:**
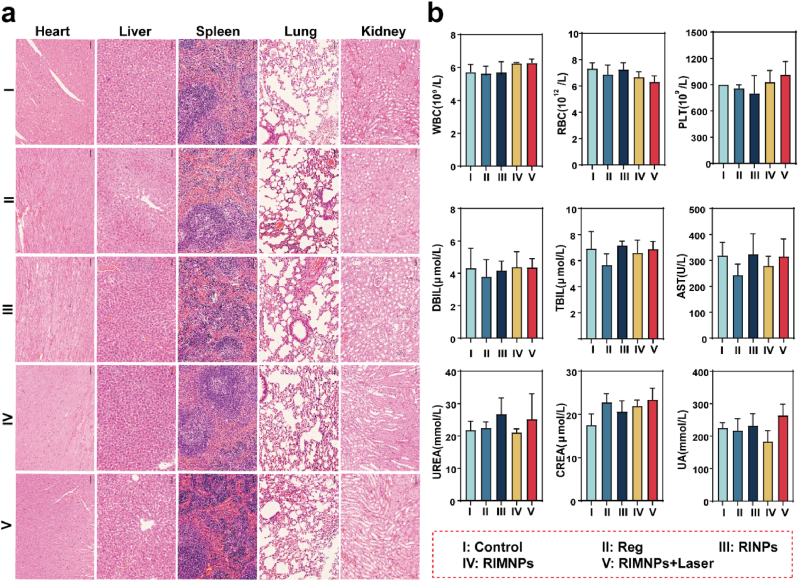
*In vivo* biosafety of RIMNPs. (a) H&E histological staining of excised organic (heart, liver, spleen, lung, and kidney) slices for PBS, Reg, RINPs, RIMNPs, and RIMNPs + Laser treatment groups, respectively. (b) Blood biochemistry analysis (WBC: white blood cells, RBC: red blood cells, PLT: platelets), liver function (TBIL: total bilirubin, DBIL: direct bilirubin, AST: alanine aminotransferase), and kidney function (UREA: urea, CREA: creatinine, UA: uric acid) in mouse serum after different treatments (n = 6).

Low side effects are a crucial prerequisite for ideal nanodrugs in cancer therapy. Consequently, we performed an in vivo biosafety evaluation using HepG2 tumor-bearing mice to investigate the systemic toxicity of RIMNPs. Hematoxylin and eosin (H&E) staining of major organs and blood biochemical analyses were conducted. As depicted in Fig. 7a, no significant damage to the nucleus or cytoplasm was observed in the major organs (heart, liver, spleen, lung, kidney) of mice from all treatment groups. Additionally, biochemical analysis using hepatic function indicators, including direct bilirubin (DBIL), alanine aminotransferase (AST), and total bilirubin (TBIL), as well as kidney function indicators, such as creatinine (CREA) and blood urea (UREA), confirmed the normal functioning of the liver and kidneys following treatment with RIMNPs (Fig. 7b). These findings indicate the excellent biosafety of the combined photothermal therapy (PTT) and Reg-based chemotherapy, affirming the potential of RIMNPs as a safe and effective treatment modality in cancer therapy.

## 4 Conclusions

In summary, we have developed cancer cell membrane bionics RIMNPs using a straightforward self-assembly strategy for targeted delivery, cancer imaging, and combined photothermal/chemotherapy. RIMNPs possess homologous targeting ability and exploit the Enhanced Permeability and Retention (EPR) effect to achieve precise and efficient tumor aggregation. The prolonged presence of RIMNPs in tumors allows for real-time fluorescence imaging with high spatial resolution and deep tissue penetration. Importantly, we demonstrated that RIMNPs exhibit volume expansion when exposed to near-infrared radiation, which directly disrupts intracellular structures and induces cancer cell death. Additionally, the excellent photothermal properties of this nanosystem provide effective photothermal therapy (PTT) capabilities that synergistically enhance the sustained chemotherapy of Reg, thereby inhibiting tumor cell proliferation, reducing tumor neovascularization, and modulating tumor-associated macrophages (TAMs). Overall, RIMNPs represent an ideal therapeutic nanomedicine for cancer-targeted imaging and synergistic photothermal/chemotherapy against hepatocellular carcinoma (HCC). They are simple to produce, safe, and highly effective, offering a promising approach to advanced cancer treatment.

## CRediT authorship contribution statement

**Weijie Wang:** Writing – original draft, Funding acquisition, Conceptualization. **Chenguang Sun:** Writing – original draft, Software, Methodology, Data curation. **Linhao Jing:** Visualization, Software, Data curation. **Yaning Xia:** Investigation, Data curation. **Shuijun Zhang:** Writing – review & editing, Supervision, Resources, Conceptualization. **Yupeng Shi:** Writing – review & editing, Supervision, Funding acquisition, Conceptualization.

## Declaration of competing interest

The authors declare that they have no known competing financial interests or personal relationships that could have appeared to influence the work reported in this paper.

## Data Availability

Data will be made available on request.

## References

[1] Vogel A., Meyer T., Sapisochin G., Salem R., Saborowski A. (2022). Hepatocellular carcinoma. Lancet.

[2] Llovet J.M., Pinyol R., Kelley R.K., El-Khoueiry A., Reeves H.L., Wang X.W., Gores G.J., Villanueva A. (2022). Molecular pathogenesis and systemic therapies for hepatocellular carcinoma. Nat. Cancer.

[3] Yang X.P., Yang C., Zhang S., Zhu A.X., Geng H.G., Bernards R., Qin W.X., Fan J., Wang C., Gao Q. (2024). Precision treatment in advanced hepatocellular carcinoma. Cancer Cell.

[4] Cheng A.L., Hsu C., Chan S.L., Choo S.P., Kudo M. (2020). Challenges of combination therapy with immune checkpoint inhibitors for hepatocellular carcinoma. J. Hepatol..

[5] Huang A., Yang X.R., Chung W.Y., Dennison A.R., Zhou J. (2020). Targeted therapy for hepatocellular carcinoma. Signal Transduct. Targeted Ther..

[6] Ou D.L., Chen C.W., Hsu C.L., Chung C.H., Feng Z.R., Lee B.S., Cheng A.L., Yang M.H., Hsu C. (2021). Regorafenib enhances antitumor immunity via inhibition of p38 kinase/Creb1/Klf4 axis in tumor-associated macrophages. J. Immunother. Cancer.

[7] Grothey A., Blay J.Y., Pavlakis N., Yoshino T., Bruix J. (2020). Evolving role of regorafenib for the treatment of advanced cancers. Cancer Treat Rev..

[8] Cousin S., Cantarel C., Guegan J.P., Gomez-Roca C., Metges J.P., Adenis A., Pernot S., Bellera C., Kind M., Auzanneau C., Le Loarer F., Soubeyran I., Bessede A., Italiano A. (2021). Regorafenib-Avelumab combination in patients with microsatellite stable colorectal cancer (REGOMUNE): a single-arm, open-label, phase II trial. Clin. Cancer Res..

[9] Bruix J., Qin S.K., Merle P., Granito A., Huang Y.H., Bodoky G., Pracht M., Yokosuka O., Rosmorduc O., Breder V., Gerolami R., Masi G., Ross P.J., Song T.Q., Bronowicki J.P., Ollivier-Hourmand I., Kudo M., Cheng A.L., Llovet J.M., Finn R.S., LeBerre M.A., Baumhauer A., Meinhardt G., Han G.H., Resorce I. (2017). Regorafenib for patients with hepatocellular carcinoma who progressed on sorafenib treatment (RESORCE): a randomised, double-blind, placebo-controlled, phase 3 trial. Lancet.

[10] Wang X.Y., Li C., Wang Y.G., Chen H.B., Zhang X.X., Luo C., Zhou W.H., Li L.L., Teng L.S., Yu H.J., Wang J.C. (2022). Smart drug delivery systems for precise cancer therapy. Acta Pharm. Sin. B.

[11] Wu Y.H., Zhu R.T., Zhou M.Y., Liu J.J., Dong K., Zhao S.F., Cao J.H., Wang W.J., Sun C.G., Wu S.T., Wang F., Shi Y.P., Sun Y.L. (2023). Homologous cancer cell membrane-camouflaged nanoparticles target drug delivery and enhance the chemotherapy efficacy of hepatocellular carcinoma. Cancer Lett..

[12] Wang D.L., Li Y., Deng X.R., Torre M., Zhang Z.P., Li X.Y., Zhang W., Cullion K., Kohane D.S., Weldon C.B. (2023). An aptamer-based depot system for sustained release of small molecule therapeutics. Nat. Commun..

[13] Zhang H.J., Pan Y.P., Hou Y., Li M.H., Deng J., Wang B.C., Hao S.L. (2024). Smart physical-based transdermal drug delivery system:towards intelligence and controlled release. Small.

[14] Han H.J., Li S., Xu M.Y., Zhong Y.Y., Fan W.J., Xu J.W., Zhou T.L., Ji J., Ye J., Yao K. (2023). Polymer- and lipid-based nanocarriers for ocular drug delivery: current status and future perspectives. Adv. Drug Deliv. Rev..

[15] Chehelgerdi M., Chehelgerdi M., Allela O.Q.B., Pecho R.D.C., Jayasankar N., Rao D.P., Thamaraikani T., Vasanthan M., Viktor P., Lakshmaiya N., Saadh M.J., Amajd A., Abo-Zaid M.A., Castillo-Acobo R.Y., Ismail A.H., Amin A.H., Akhavan-Sigari R. (2023). Progressing nanotechnology to improve targeted cancer treatment: overcoming hurdles in its clinical implementation. Mol. Cancer.

[16] Guo C.Q., Wu M.Y., Guo Z.F., Zhang R.P., Wang Z.J., Peng X., Dong J.X., Sun X., Zhang Z.R., Xiao P.H., Gong T. (2023). Hypoxia-responsive golgi-targeted prodrug assembled with anthracycline for improved antitumor and antimetastasis efficacy. ACS Nano.

[17] Zhou X.Q., Wang P.Y., Ramu V., Zhang L.Y., Jiang S.H., Li X.Z., Abyar S., Papadopoulou P., Shao Y., Bretin L., Siegler M.A., Buda F., Kros A., Fan J.L., Peng X.J., Sun W., Bonnet S. (2023). In vivo metallophilic self-assembly of a light-activated anticancer drug. Nat. Chem..

[18] Zhang D.Y., Liang Y.Q., Wang M.C., Younis M.R., Yi H.X., Zhao X.Y., Chang J.S., Zheng Y., Guo W.S., Yu X.Y. (2023). Self-assembled carrier-free nanodrugs for starvation therapy-amplified photodynamic therapy of cancer. Adv. Healthcare Mater..

[19] Liu L.H., Zhang X.Z. (2022). Carrier-free nanomedicines for cancer treatment. Prog. Mater. Sci..

[20] Zuo J.H., Gao X., Xiao J.R., Cheng Y.Y. (2023). Carrier-free supramolecular nanomedicines assembled by small-molecule therapeutics for cancer treatment. Chin. Chem. Lett..

[21] Zhang X.Y., Xu X.L., Liu H.M., Ni N.Y., Liu S.Q., Gong Y.F., Ma G.Q., Song L.L., Meng Q.W., Fan Q., Sun X. (2023). CCR2-overexpressing biomimetic carrier-free nanoplatform for enhanced cascade ferroptosis tumor therapy. Acta Biomater..

[22] Wang Z.L., Wang Q., Cao H.M., Wang Z.Y., Wang D.Y., Liu J.J., Gao T.X., Ren C.H., Liu J.F. (2024). Mitochondrial localized in situ self-assembly reprogramming tumor immune and metabolic microenvironment for enhanced cancer therapy. Adv. Mater..

[23] Yan Z.L., Liu Y.F., Zhao L.C., Hu J.X., Du Y.M., Peng X.X., Liu Z.B. (2023). *In situ* stimulus-responsive self-assembled nanomaterials for drug delivery and disease treatment. Mater. Horiz..

[24] Liang X.Y., Zhang Y., Zhou J., Bu Z.T., Liu J.J., Zhang K. (2022). Tumor microenvironment-triggered intratumoral in situ construction of theranostic supramolecular self-assembly. Coord. Chem. Rev..

[25] Lee H.P., Gaharwar A.K. (2020). Light-responsive inorganic biomaterials for biomedical applications. Adv. Sci..

[26] Xu F., Feringa B.L. (2023). Photoresponsive supramolecular polymers: from light-controlled small molecules to smart materials. Adv. Mater..

[27] Jiang L.D., Chen D.Y., Jin Z.K., Xia C., Xu Q.Q., Fan M.J., Dai Y.L., Liu J., Li Y.P., He Q.J. (2022). Light-triggered nitric oxide release and structure transformation of peptide for enhanced intratumoral retention and sensitized photodynamic therapy. Bioact. Mater..

[28] Yu Y.J., Tang D.S., Liu C.Y., Zhang Q., Tang L., Lu Y.F., Xiao H.H. (2022). Biodegradable polymer with effective near-infrared-II absorption as a photothermal agent for deep tumor therapy. Adv. Mater..

[29] Ge R.L., Yan P.N., Liu Y., Li Z.S., Shen S.Q., Yu Y. (2023). Recent advances and clinical potential of near infrared photothermal conversion materials for photothermal hepatocellular carcinoma therapy. Adv. Funct. Mater..

[30] Zhou Z.J., Yan Y., Hu K.W., Zou Y., Li Y.W., Ma R., Zhang Q., Cheng Y.Y. (2017). Autophagy inhibition enabled efficient photothermal therapy at a mild temperature. Biomaterials.

[31] Geng S.A., Guo M.Q., Zhan G.T., Shi D.W., Shi L.Y., Gan L., Zhao Y.B., Yang X.L. (2023). NIR-triggered ligand-presenting nanocarriers for enhancing synergistic photothermal-chemotherapy. J. Contr. Release.

[32] Bunschoten A., Buckle T., Kuil J., Luker G.D., Luker K.E., Nieweg O.E., van Leeuwen F.W.B. (2012). Targeted non-covalent self-assembled nanoparticles based on human serum albumin. Biomaterials.

[33] Zheng M.C., Zhang J.J., Deng C.T., Chen L., Zhang H., Xin J.Q., Aras O., Zhou M.J., An F.F., Ren Y. (2024). The collaborated assembly of hydrophobic curcumin and hydrophilic cyanine dye into nanocolloid for synergistic chemo-photothermal cancer therapy. Mater. Des..

[34] Shamay Y., Shah J., Isik M., Mizrachi A., Leibold J., Tschaharganeh D.F., Roxbury D., Budhathoki-Uprety J., Nawaly K., Sugarman J.L., Baut E., Neiman M.R., Dacek M., Ganesh K.S., Johnson D.C., Sridharan R., Chu K.L., Rajasekhar V.K., Lowe S.W., Chodera J.D., Heller D.A. (2018). Quantitative self-assembly prediction yields targeted nanomedicines. Nat. Mater..

[35] Hu C.M.J., Zhang L., Aryal S., Cheung C., Fang R.H., Zhang L.F. (2011). Erythrocyte membrane-camouflaged polymeric nanoparticles as a biomimetic delivery platform. Proc. Natl. Acad. Sci. USA.

[36] Fang R.H., Kroll A.V., Gao W.W., Zhang L.F. (2018). Cell membrane coating nanotechnology. Adv. Mater..

[37] Luk B.T., Zhang L.F. (2015). Cell membrane-camouflaged nanoparticles for drug delivery. J. Contr. Release.

[38] Lei W., Yang C., Wu Y., Ru G.Q., He X.L., Tong X.M., Wang S.B. (2022). Nanocarriers surface engineered with cell membranes for cancer targeted chemotherapy. J. Nanobiotechnol..

[39] Liu L.Z., Bai X., Martikainen M.V., Kårlund A., Roponen M., Xu W.J., Hu G.Q., Tasciotti E., Lehto V.P. (2021). Cell membrane coating integrity affects the internalization mechanism of biomimetic nanoparticles. Nat. Commun..

[40] Gao D., Shi Y.P., Ni J.H., Chen S.J., Wang Y., Zhao B., Song M.L., Guo X.Q., Ren X.C., Zhang X.C., Tian Z.M., Yang Z. (2022). NIR/MRI-Guided oxygen-independent carrier-free anti-tumor nano-theranostics. Small.

[41] Zhou M.Y., Wang Y.F., Xia Y.N., Li Y.H., Bao J.F., Zhang Y., Cheng J.L., Shi Y.P. (2024). MRI-guided cell membrane-camouflaged bimetallic coordination nanoplatform for combined tumor phototherapy. Mater. Today Bio.

[42] Liu P.L., Huang Y., Zhan C.Y., Zhang F., Deng C.S., Jia Y.M., Wan T., Wang S., Li B.W. (2023). Tumor-overexpressed enzyme responsive amphiphiles small molecular self-assembly nano-prodrug for the chemo-phototherapy against non-small-cell lung cancer. Mater. Today Bio.

[43] Lu L., Wang K., Lin C.C., Yang W.H., Duan Q.J., Li K., Cai K.Y. (2021). Constructing nanocomplexes by multicomponent self-assembly for curing orthotopic glioblastoma with synergistic chemo-photothermal therapy. Biomaterials.

[44] Chen C., Wu Y., Wang S.T., Berisha N., Manzari M.T., Vogt K., Gang O.L., Heller D.A. (2023). Fragment-based drug nanoaggregation reveals drivers of self-assembly. Nat. Commun..

[45] Saad W.S., Prud'homme R.K. (2016). Principles of nanoparticle formation by flash nanoprecipitation. Nano Today.

[46] Long K.Q., Wang Y.F., Lv W., Yang Y., Xu S.T., Zhan C.Y., Wang W.P. (2022). Photoresponsive prodrug-dye nanoassembly for in-situ monitorable cancer therapy. Bioeng. Transl. Med..

[47] Han X.J., Gong C.A., Yang Q.R., Zheng K.L., Wang Z., Zhang W. (2024). Biomimetic nano-drug delivery system: an emerging platform for promoting tumor treatment. Int. J. Nanomed..

[48] Shen X.D., Pan D.Y., Gong Q.Y., Gu Z.W., Luo K. (2024). Enhancing drug penetration in solid tumors via nanomedicine: evaluation models, strategies and perspectives. Bioact. Mater..

[49] Wang H.B., Wang X.Y., Zhang X., Xu W.H. (2024). The promising role of tumor-associated macrophages in the treatment of cancer. Drug Resist. Updates.

[50] Zhang X., Li G., Yin J., Pan W., Li Y., Li N., Tang B. (2024). Reprogramming tumor-associated macrophages with a Se-based core-satellite nanoassembly to enhance cancer immunotherapy. Nano Lett..

[51] Kim S.W., Kim C.W., Moon Y.A., Kim H.S. (2024). Reprogramming of tumor-associated macrophages by metabolites generated from tumor microenvironment. Anim. Cell Syst..

[52] Qi M.L., Ren X., Li W., Sun Y., Sun X.L., Li C.Y., Yu S.Y., Xu L., Zhou Y.M., Song S.Y., Dong B., Wang L. (2022). NIR responsive nitric oxide nanogenerator for enhanced biofilm eradication and inflammation immunotherapy against periodontal diseases. Nano Today.

[53] Teufel M., Seidel H., Köchert K., Meinhardt G., Finn R.S., Llovet J.M., Bruix J. (2019). Biomarkers associated with response to regorafenib in patients with hepatocellular carcinoma. Gastroenterology.

