# Supplementary material for: Light-driven secondary structural remodeling in biomimetic nanosystem to enhance tumor chemo-phototherapy
Source: Mater Today Bio. 2025 Jun 6;33:101955. doi: 10.1016/j.mtbio.2025.101955 (PMC12178714; doi:10.1016/j.mtbio.2025.101955)
Supplement: Multimedia component 1 [file mmc1.docx]

**Light-driven secondary structural remodeling in biomimetic nanosystem to enhance tumor chemo-phototherapy**

Weijie Wang^1#^, Chenguang Sun^1#^, Linhao, Jing^1^, Yaning Xia^2^, Shuijun Zhang^1*^, Yupeng Shi^2*^

^1^ Department of Hepatobiliary and Pancreatic Surgery, The First Affiliated Hospital of Zhengzhou University, Zhengzhou,450052, China.

^2^ Department of MRI, The First Affiliated Hospital of Zhengzhou University, Zhengzhou, 450052, China.

* Corresponding author.

[shiyup@zzu.edu.cn](mailto:shiyup@zzu.edu.cn) (Y. Shi)

#These authors have contributed equally to this work and share first authorship.


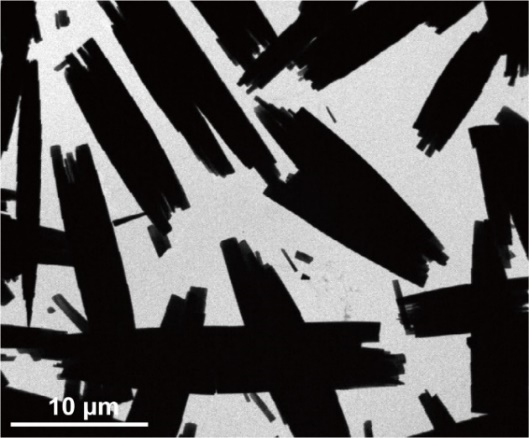


**Figure S1.** TEM of Regorafenib in aqueous solution.


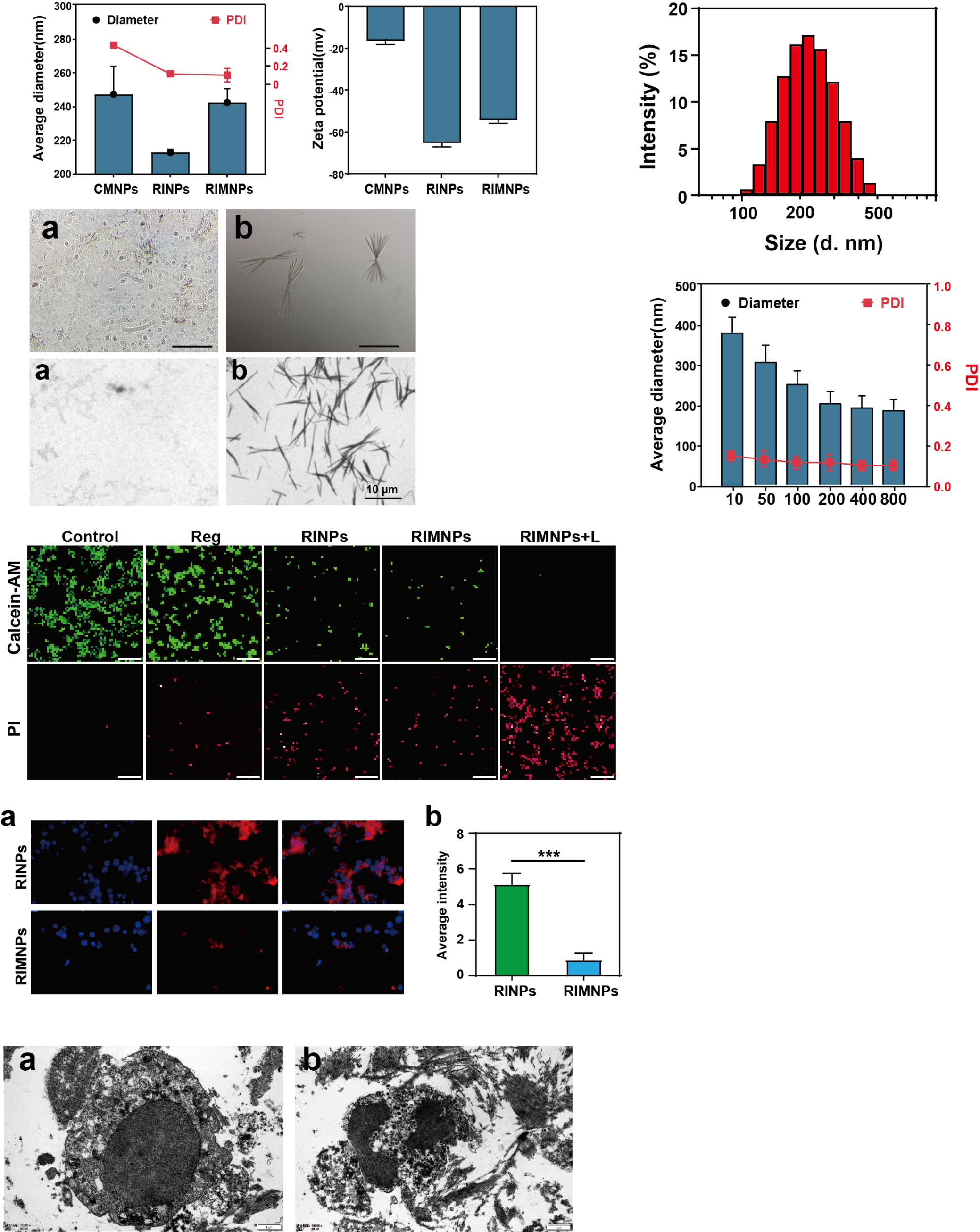


**Figure S2.** Size and PDI of Reg/IR783 NPs when prepared with different concentrations of IR783 solutions (n = 3).


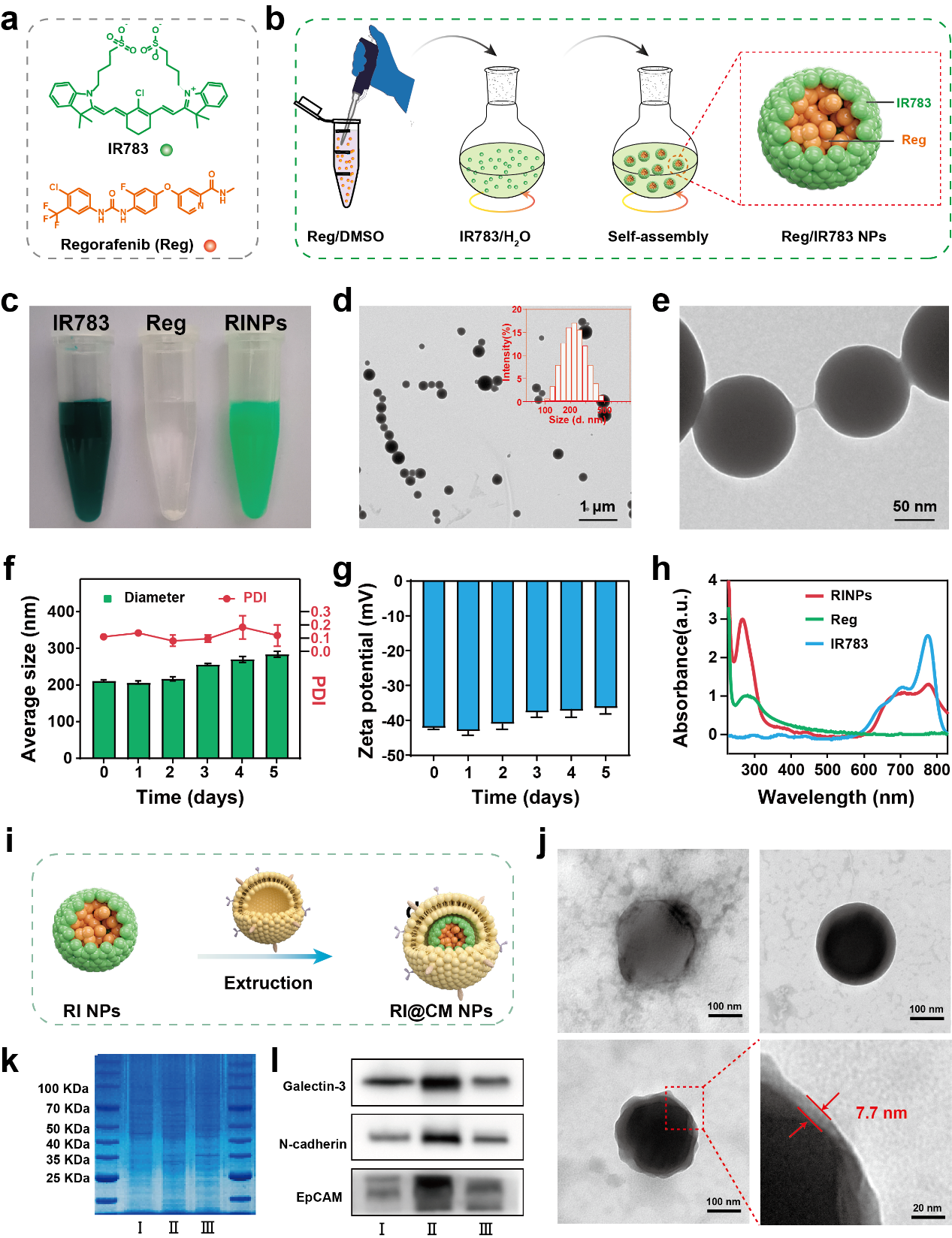


**Figure S3.** Hydrodynamic diameter, polydispersity of RINPs in deionized water for 5 days (n = 3).


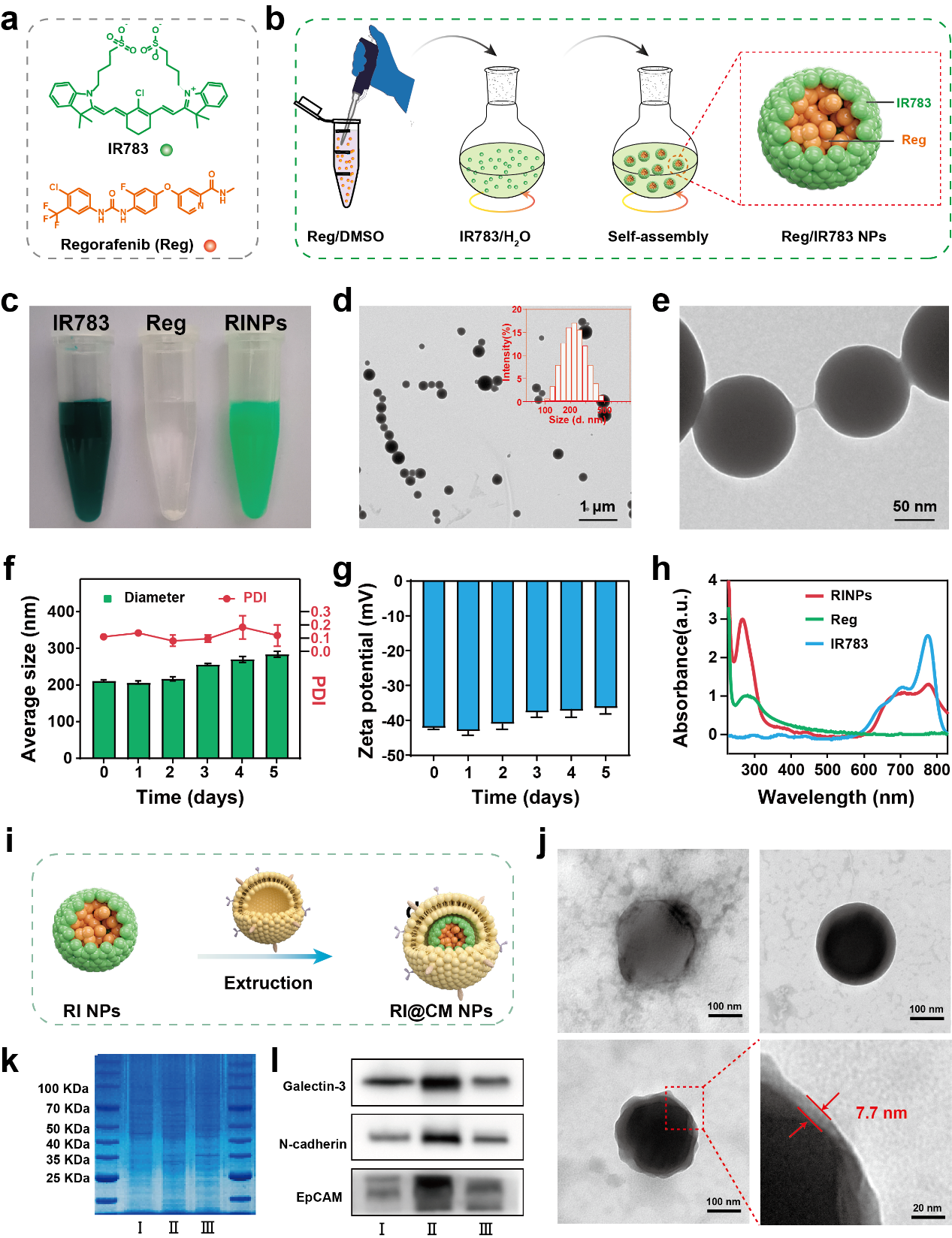


**Figure S4.** Zeta potential of RINPs in deionized water for 5 days (n = 3).

**Table S1.** Loading capacity and encapsulation efficiency of IR783 and Reg in RINPs.

|  | **Feed weight (μg)** | **Recovery weight (μg)** | **Loading Capacity** | **Encapsulation Efficiency** |
| --- | --- | --- | --- | --- |
| **Reg** | 100 μg | 85.24 | 99.67% | 85.24% |
| **IR783** | 60 μg | 0.28 | 0.33 % | 0.47% |


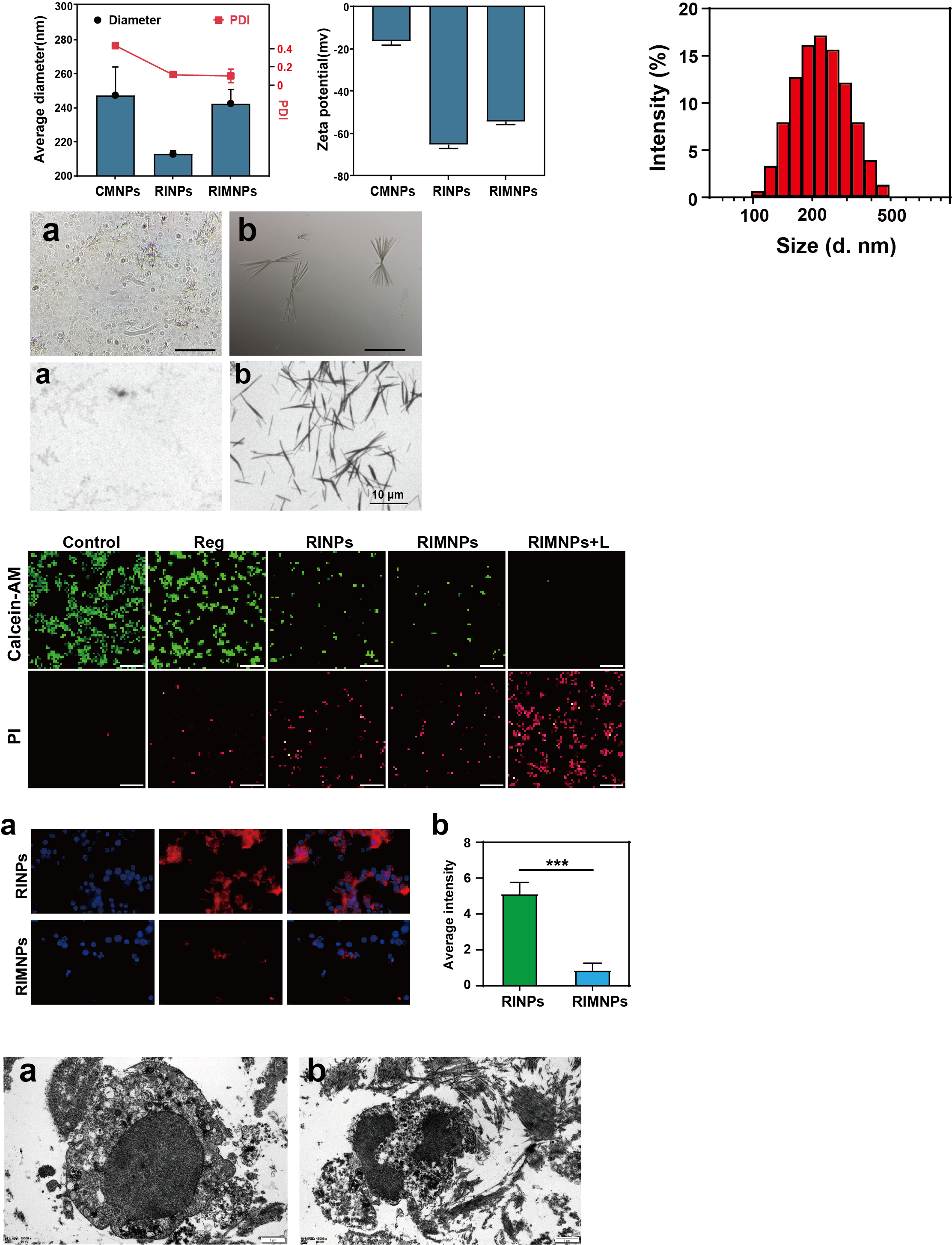


**Figure S5.** Hydrodynamic size, polydispersity of CMNPs, RINPs, and RIMNPs in deionized water (n = 3).


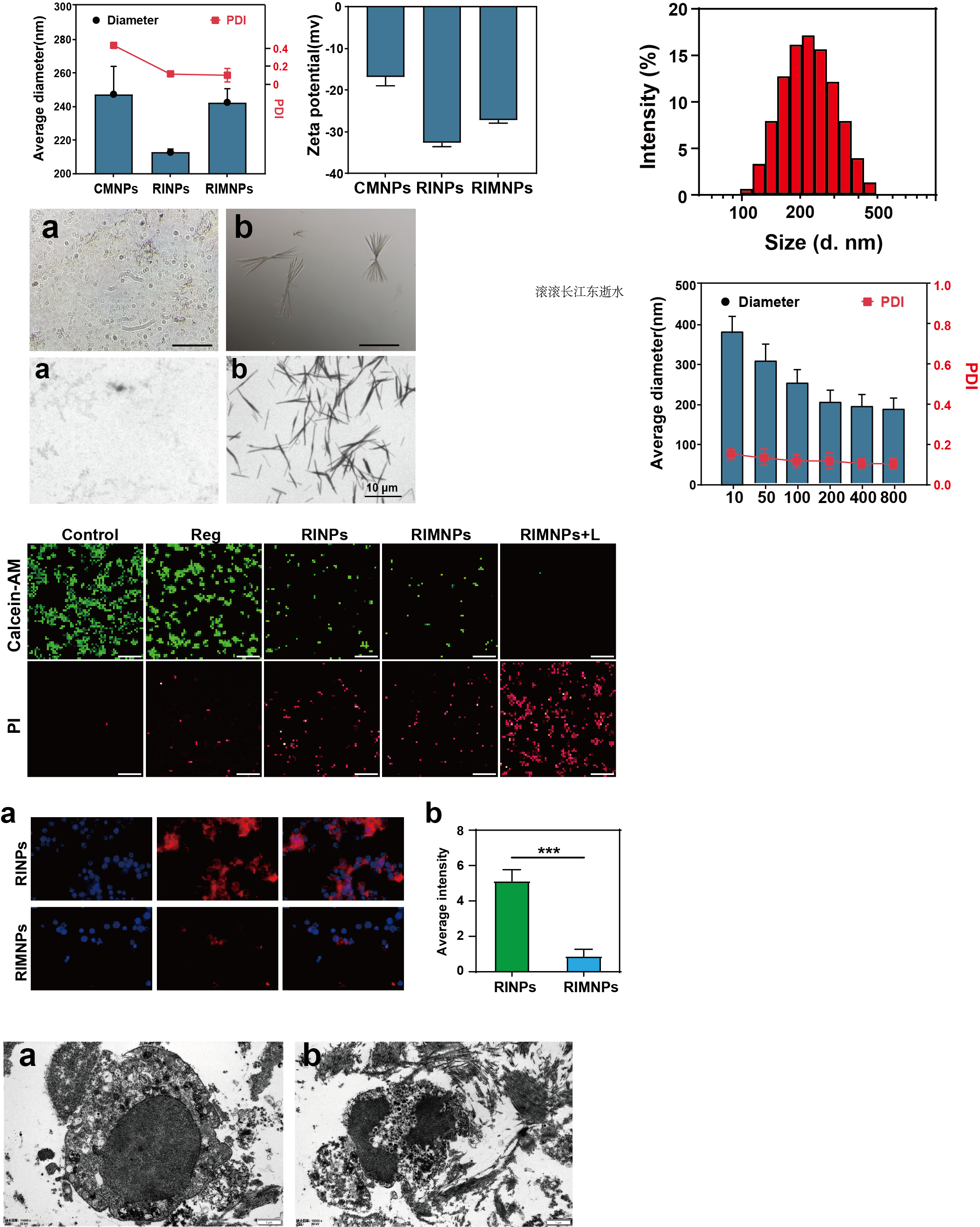


**Figure S6.** Zeta-potential of CMNPs, RINPs, and RIMNPs in deionized water (n = 3).


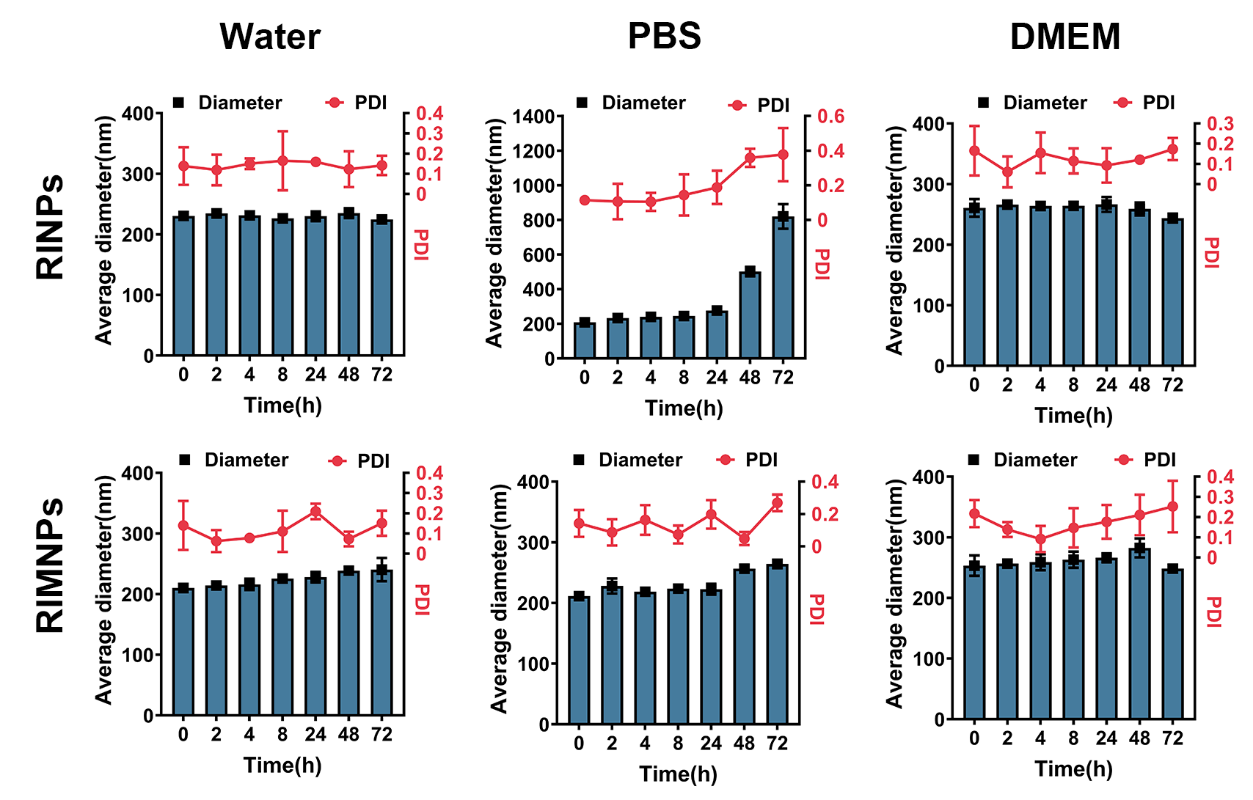

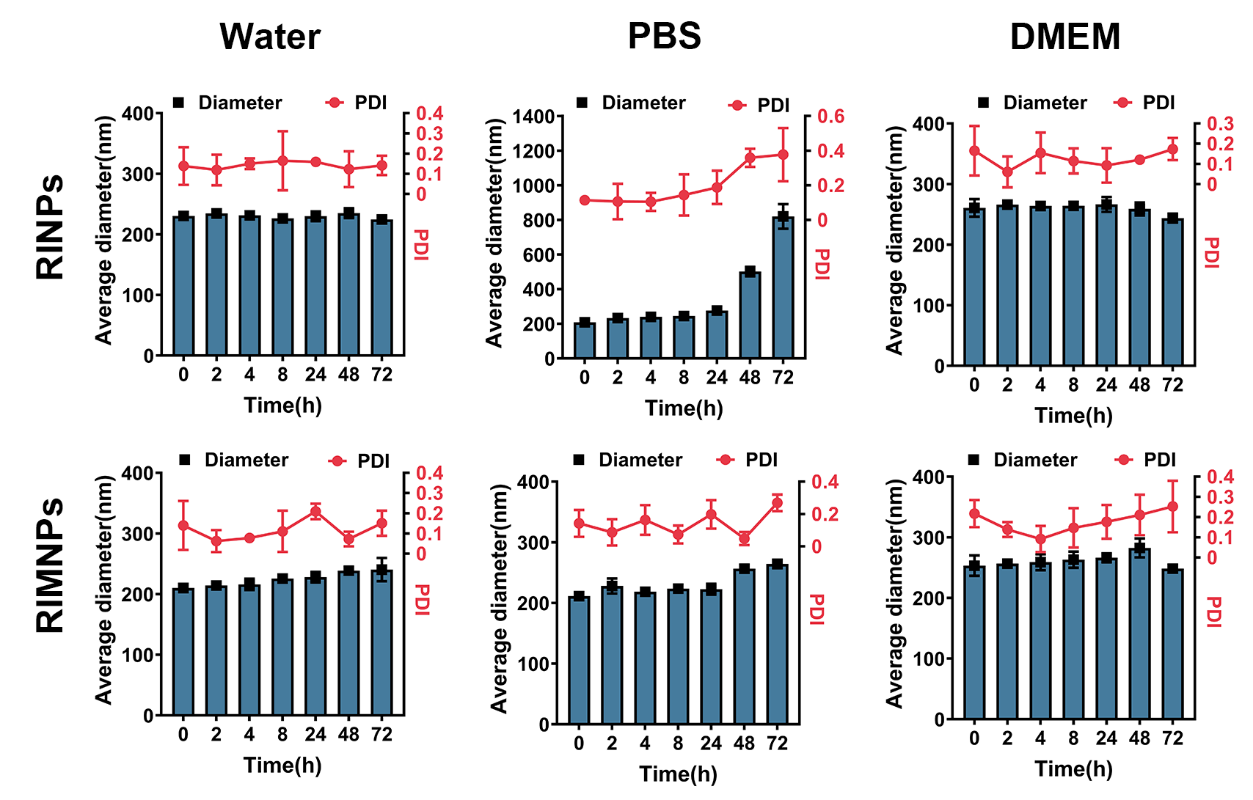


**Figure S7.** Hydrodynamic size and polydispersity of RIMNPs in deionized water and DMEM over 3 days monitored by DLS (n = 3). Data represented as mean ± SD.


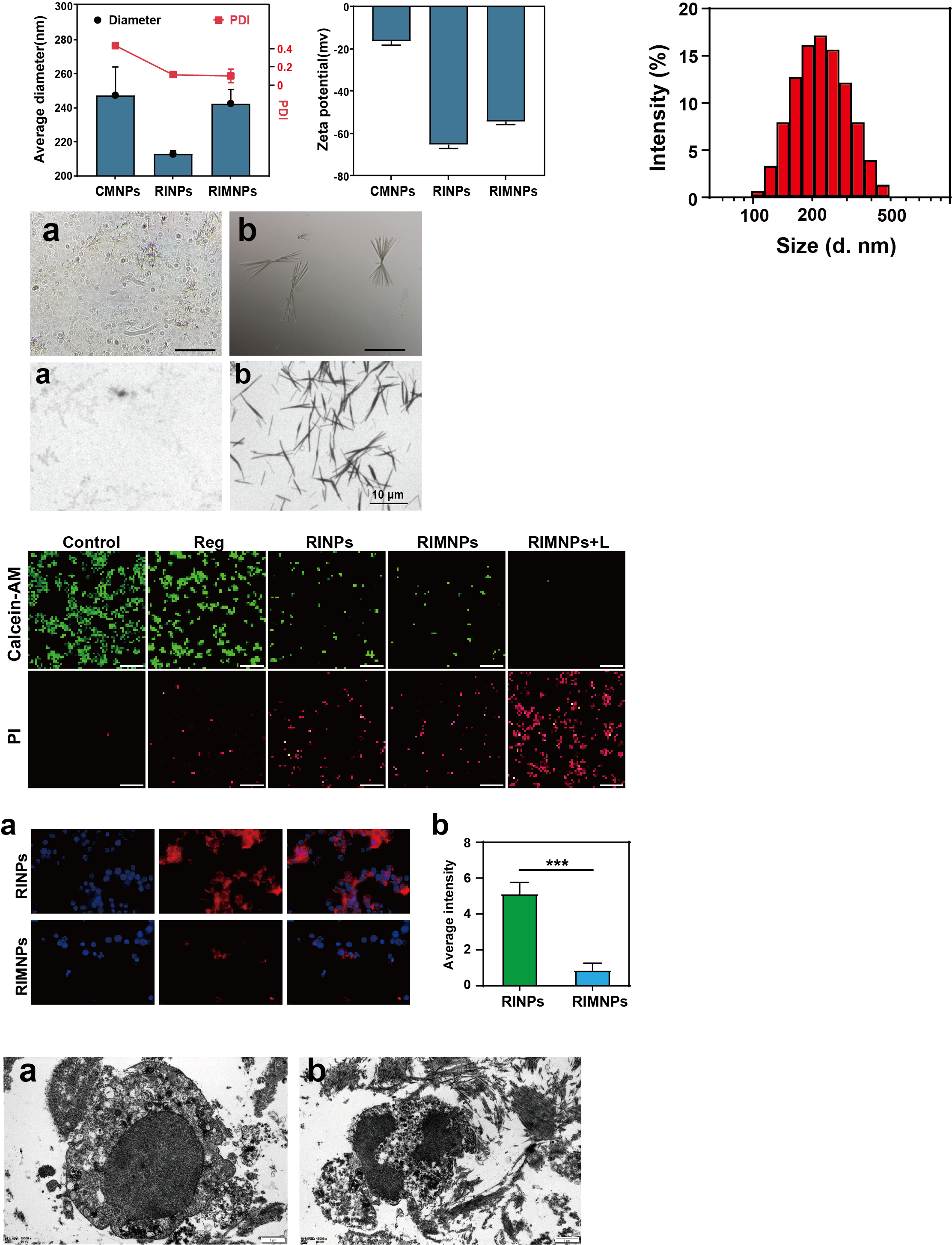


**Figure S8.** Microscope images of RINPs before and after 808 nm laser irradiation at 1.0 W/cm^2^ for 5 min (scale bar = 20 μm).


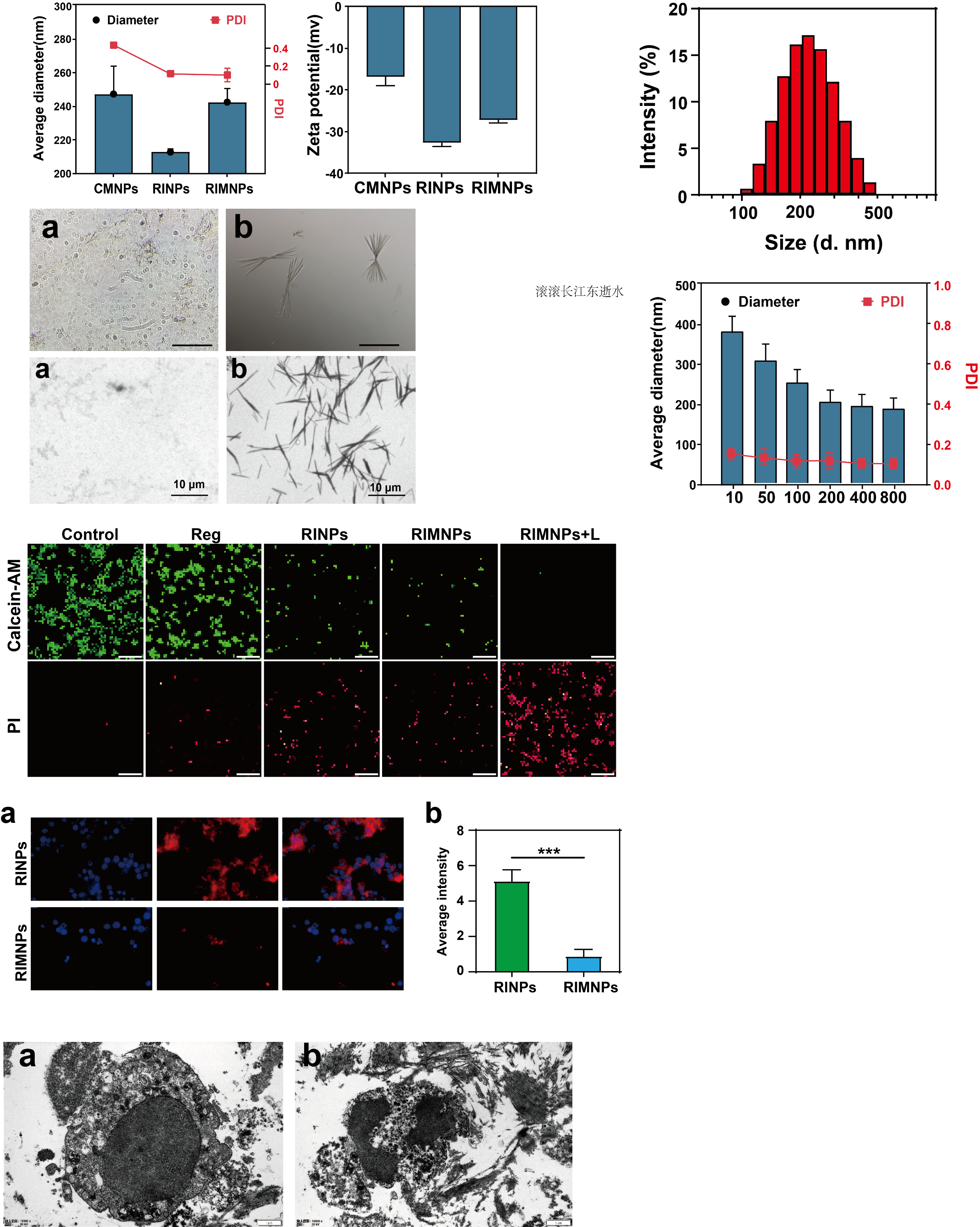


**Figure S9.** TEM images of RINPs before and after 808 nm laser irradiation at 1.0 W/cm^2^ for 5 min (scale bar = 10 μm).


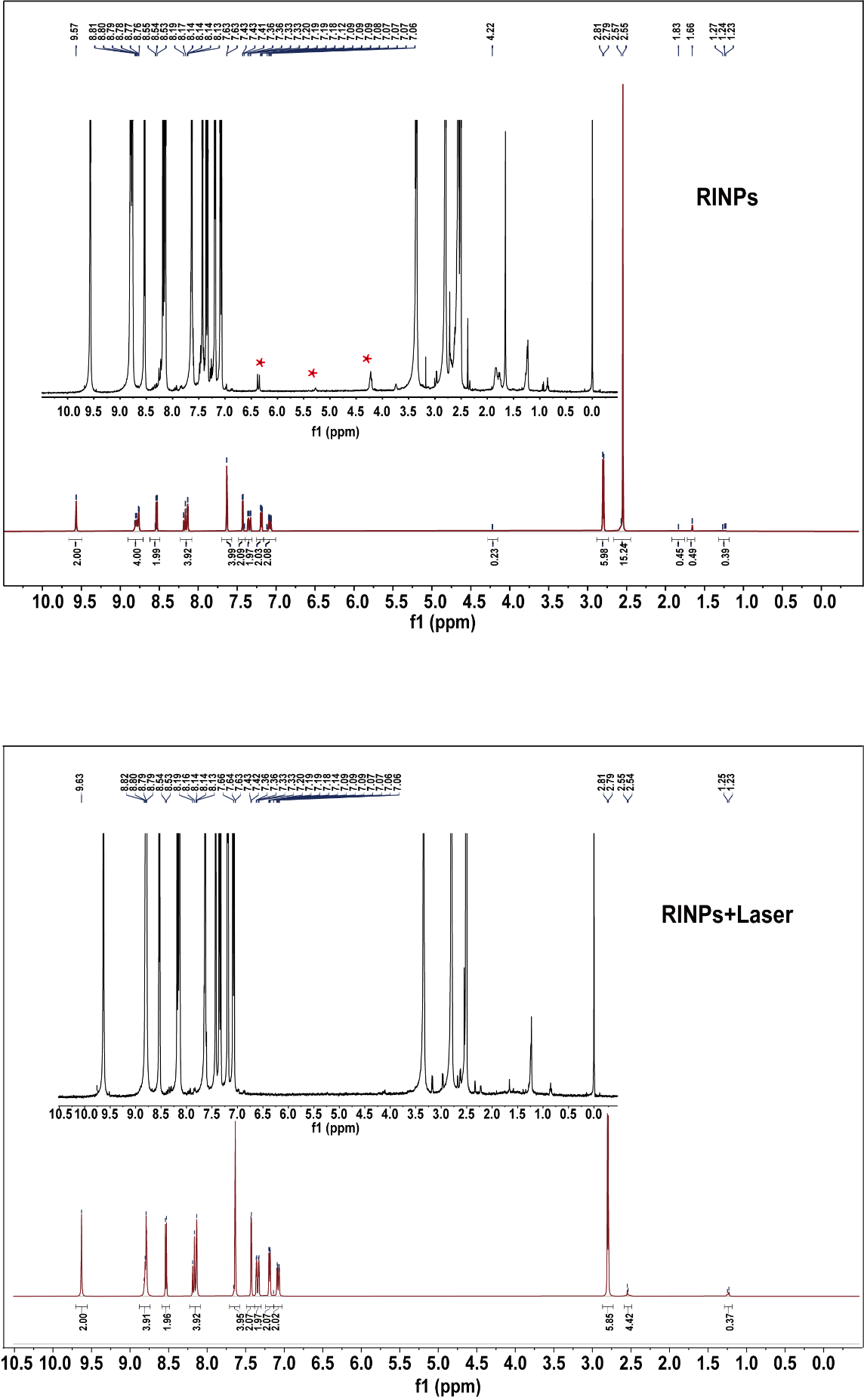


**Figure S10.** ^1^H NMR spectrum of RINPs using DMSO-d_6_ as a solvent.


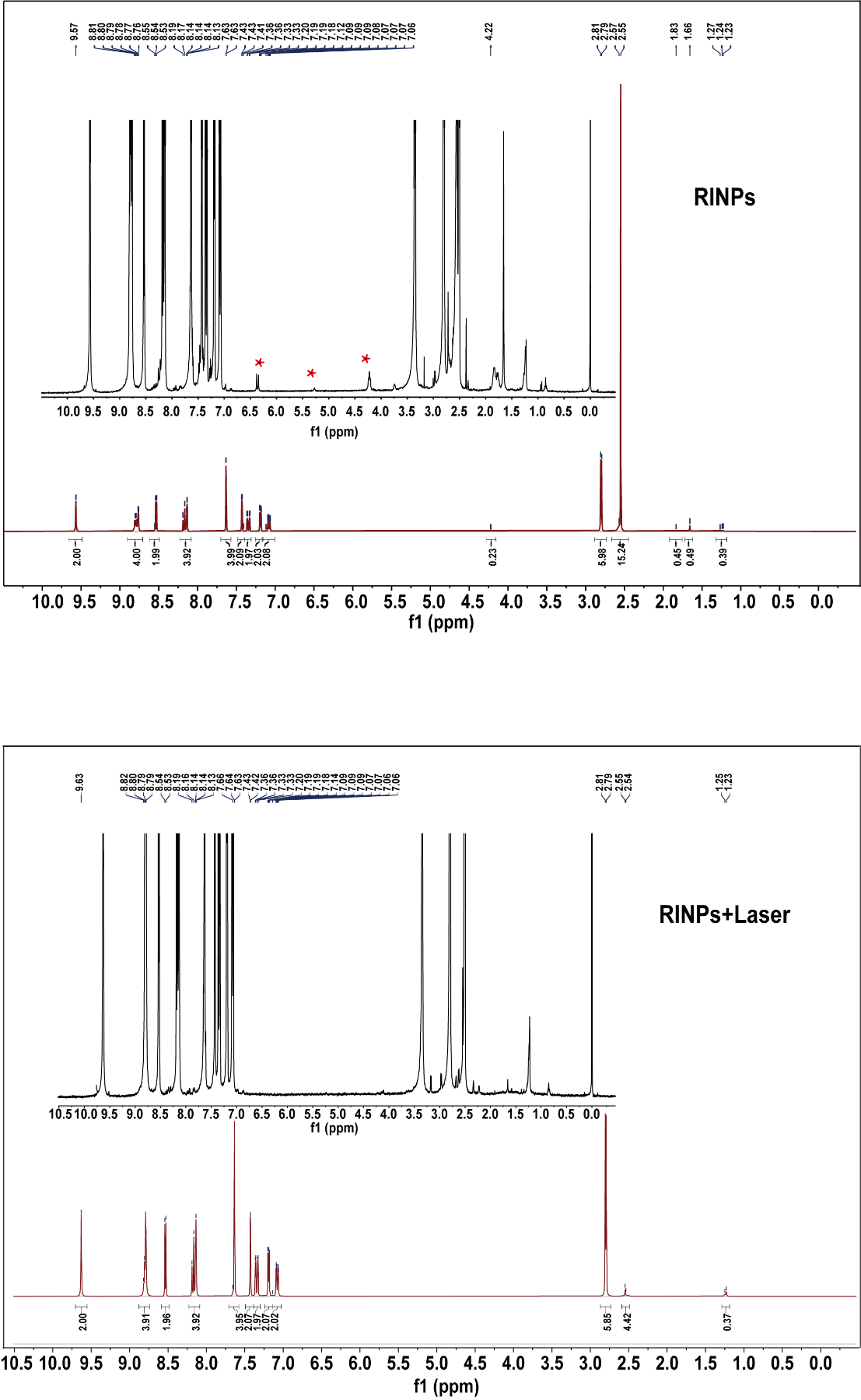


**Figure S11.** ^1^H NMR spectrum of RINPs before and after irradiation with 808 nm laser for 5 min and using DMSO-d_6_ as a solvent.


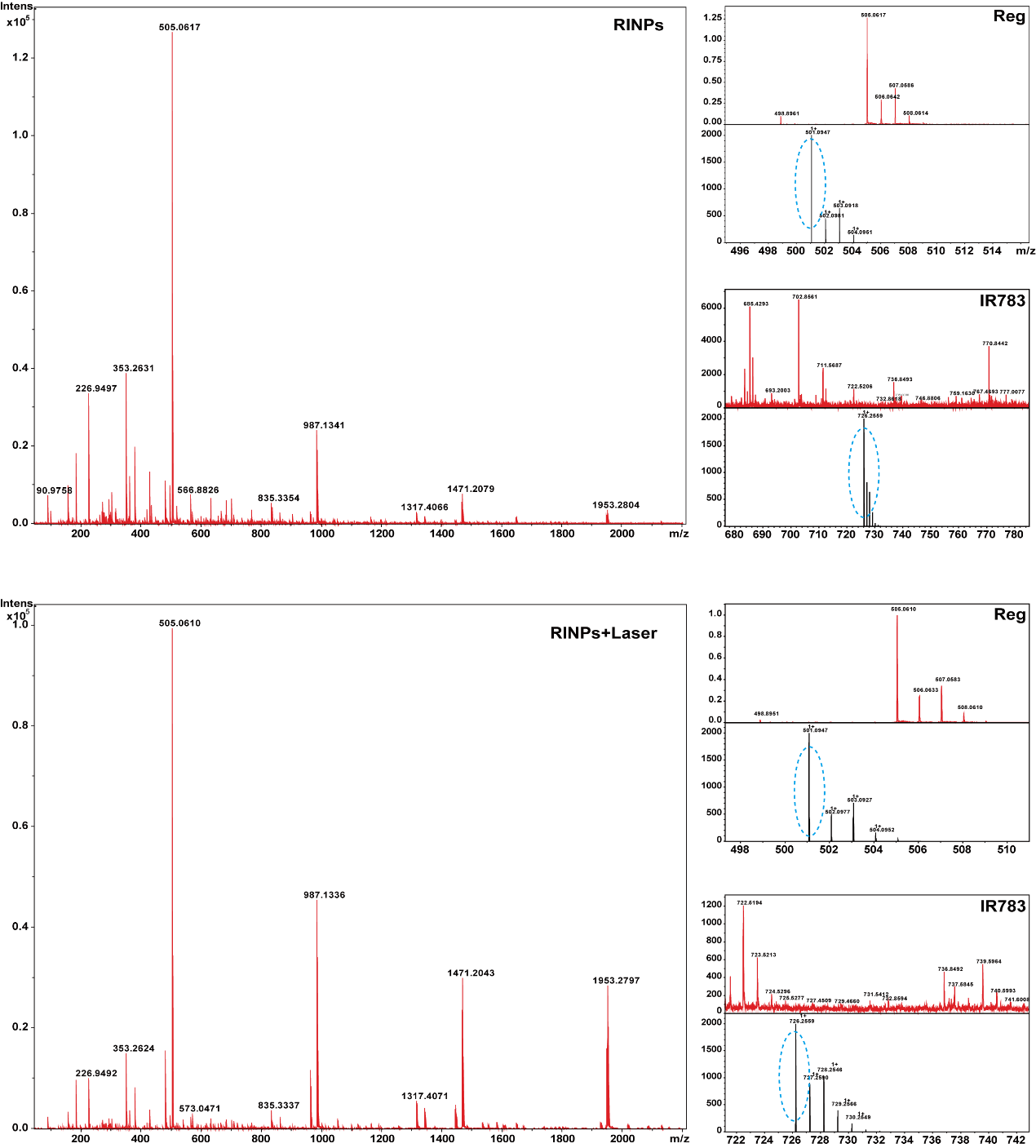


**Figure S12.** HR-MS spectrum of RINPs.


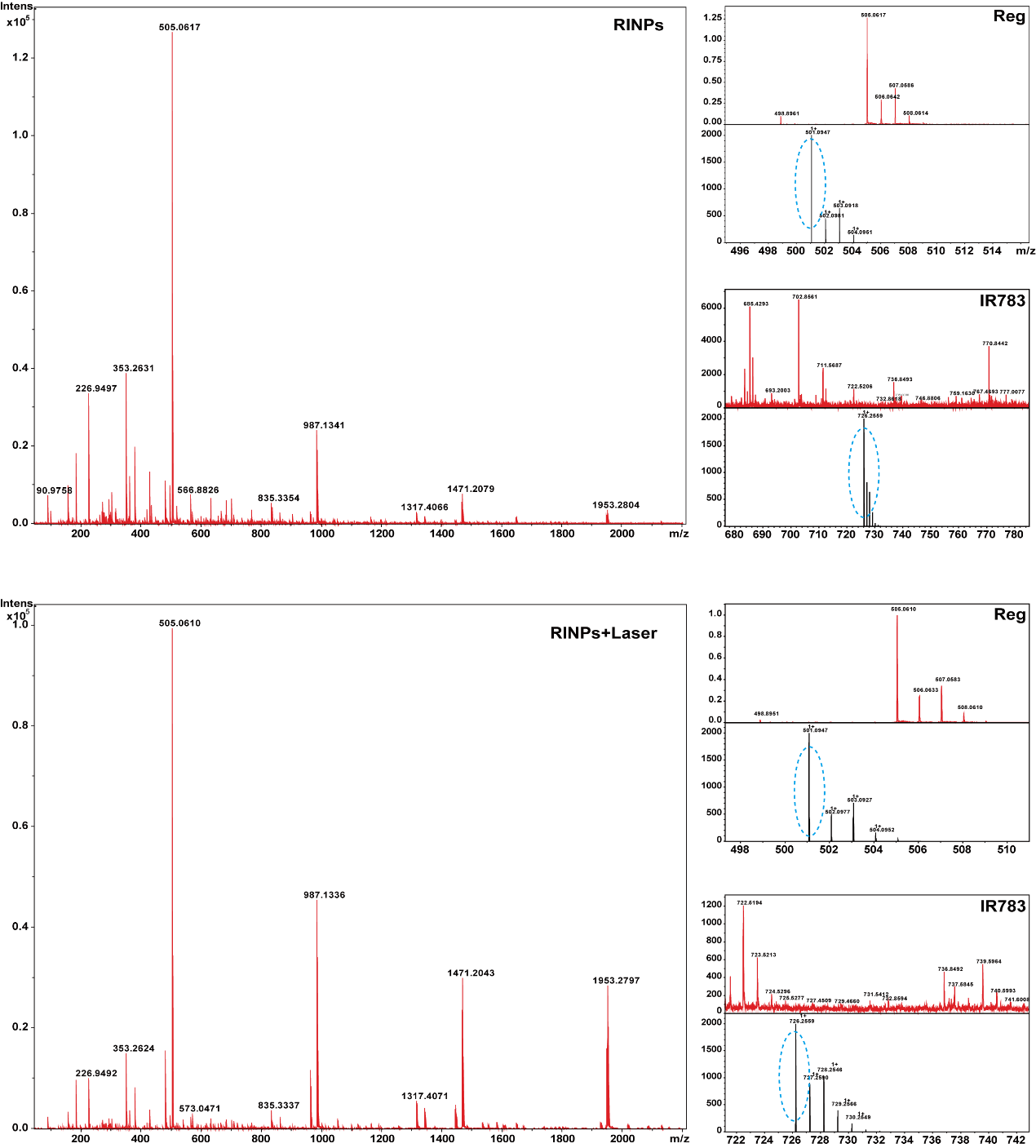


**Figure S13.** HR-MS spectrum of RINPs and the product from the reaction of RINPs with laser irradiation for 5 min.


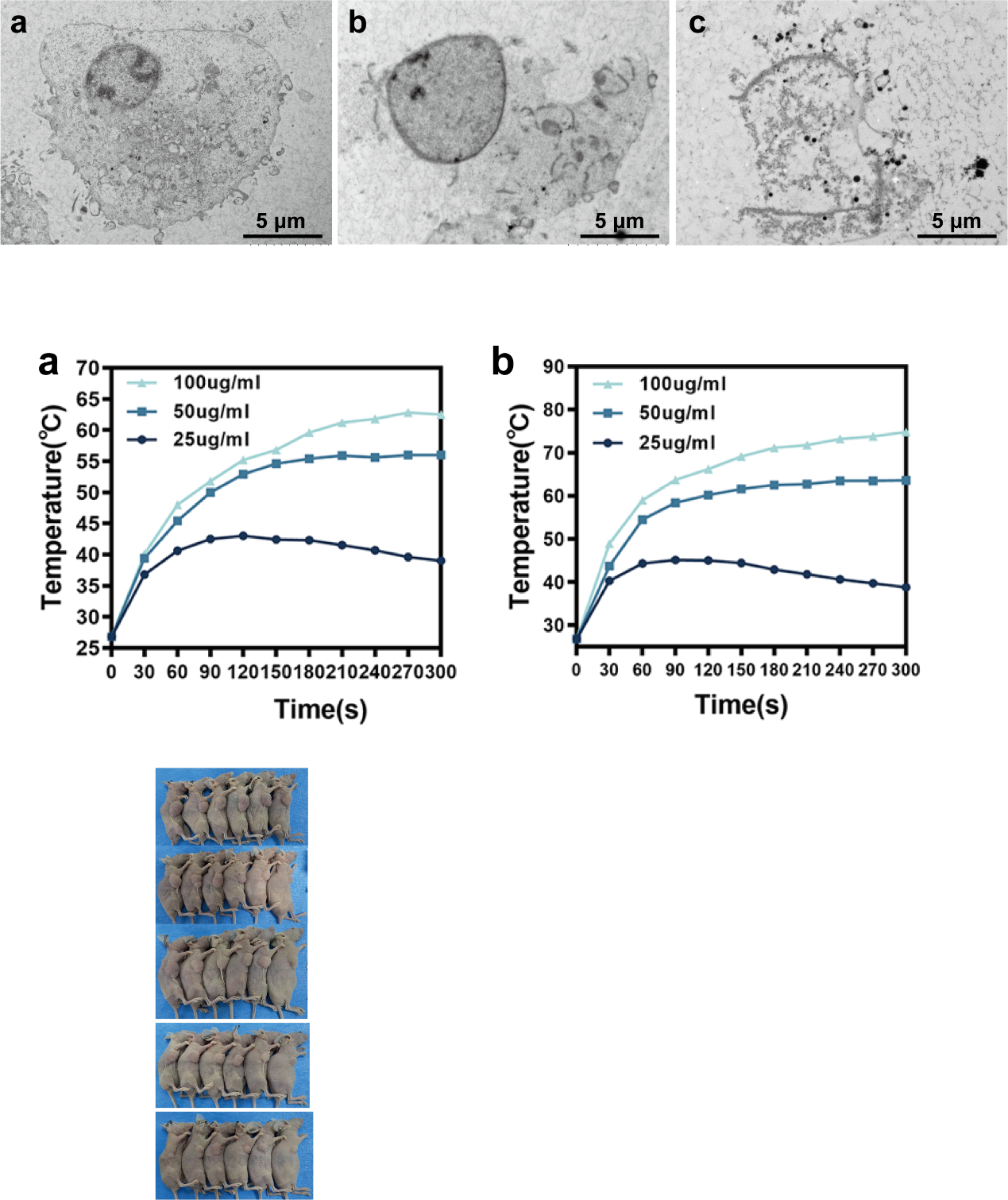


**Figure S14.** The heating curve of RINPs with different concentrations (25, 50, and 100 μg/mL^-1^) under different 808 nm laser irradiation (1.0 and 1.5 W/cm^2^).


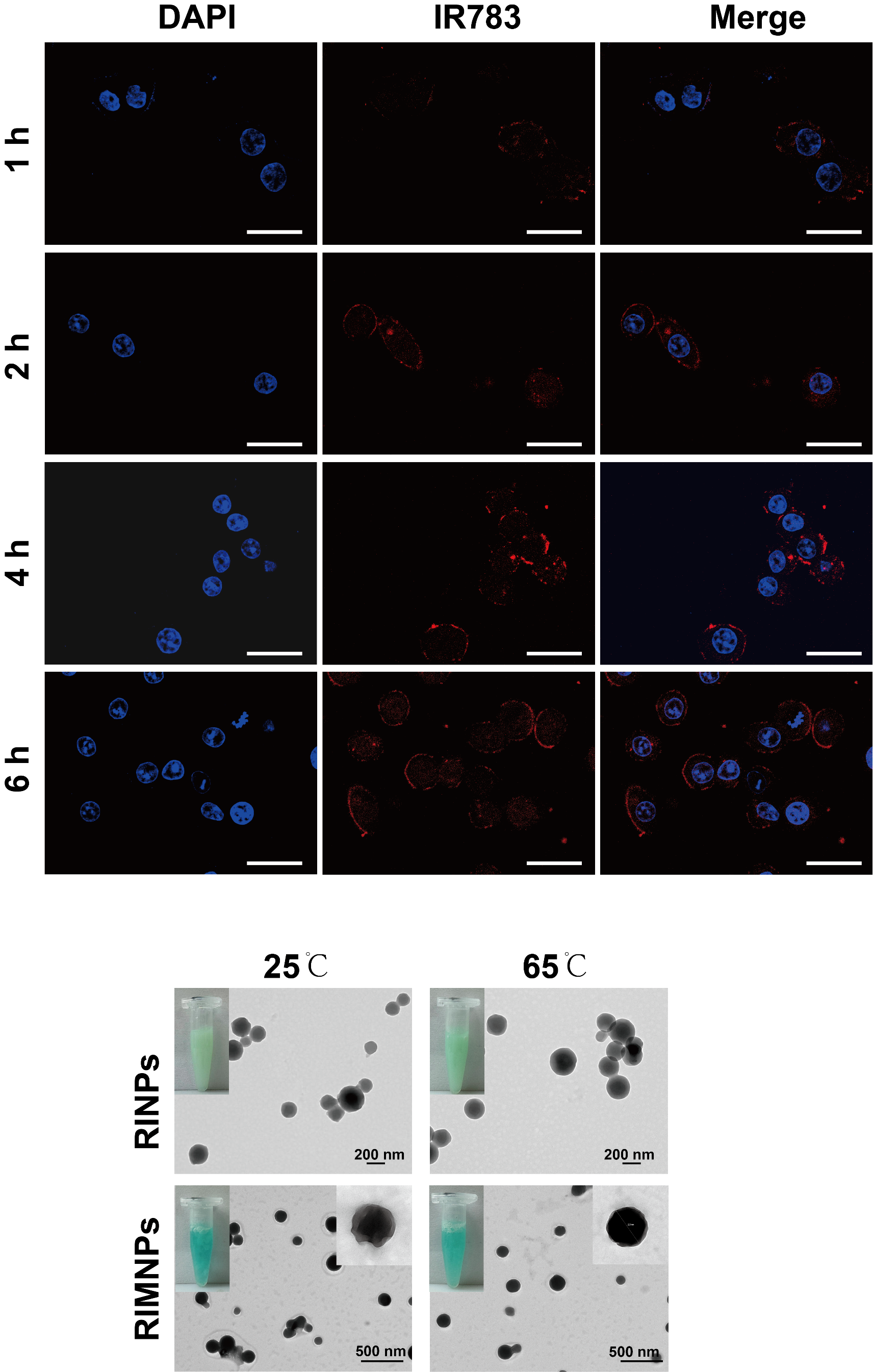


**Figure S15.** The color and morphology of RINPs and RIMNPs at 25°C and after incubation at 65°C for 10 minutes.


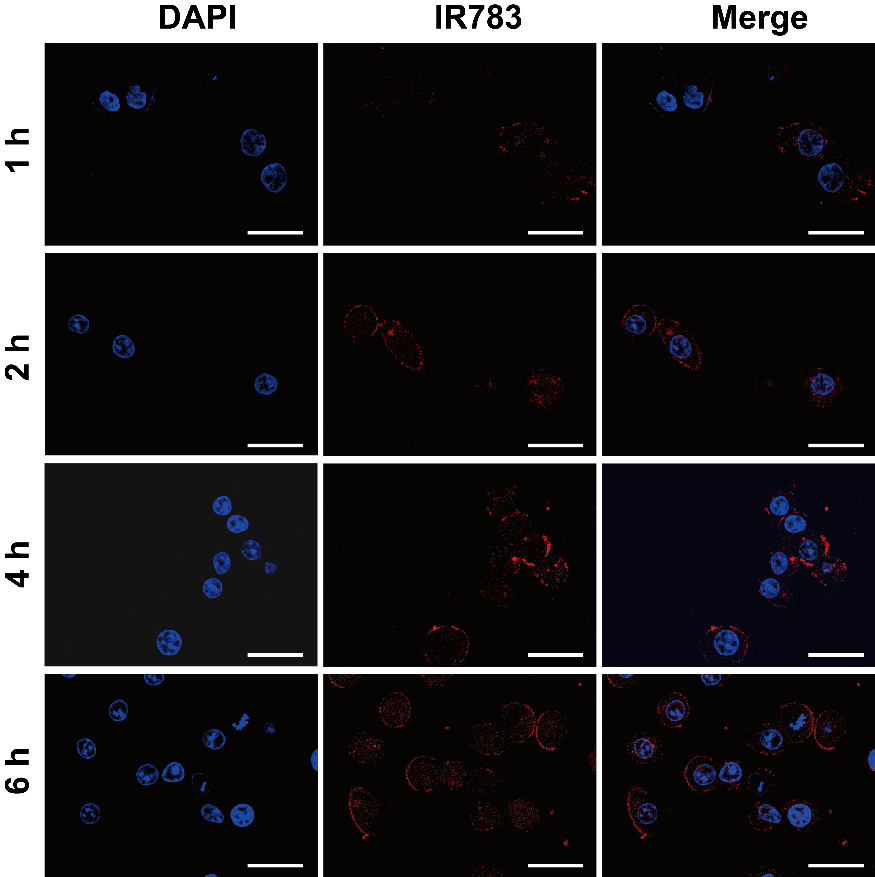


**Figure S16.** Fluorescence imaging analysis of uptake from HepG2 cells against RIMNPs at different times.


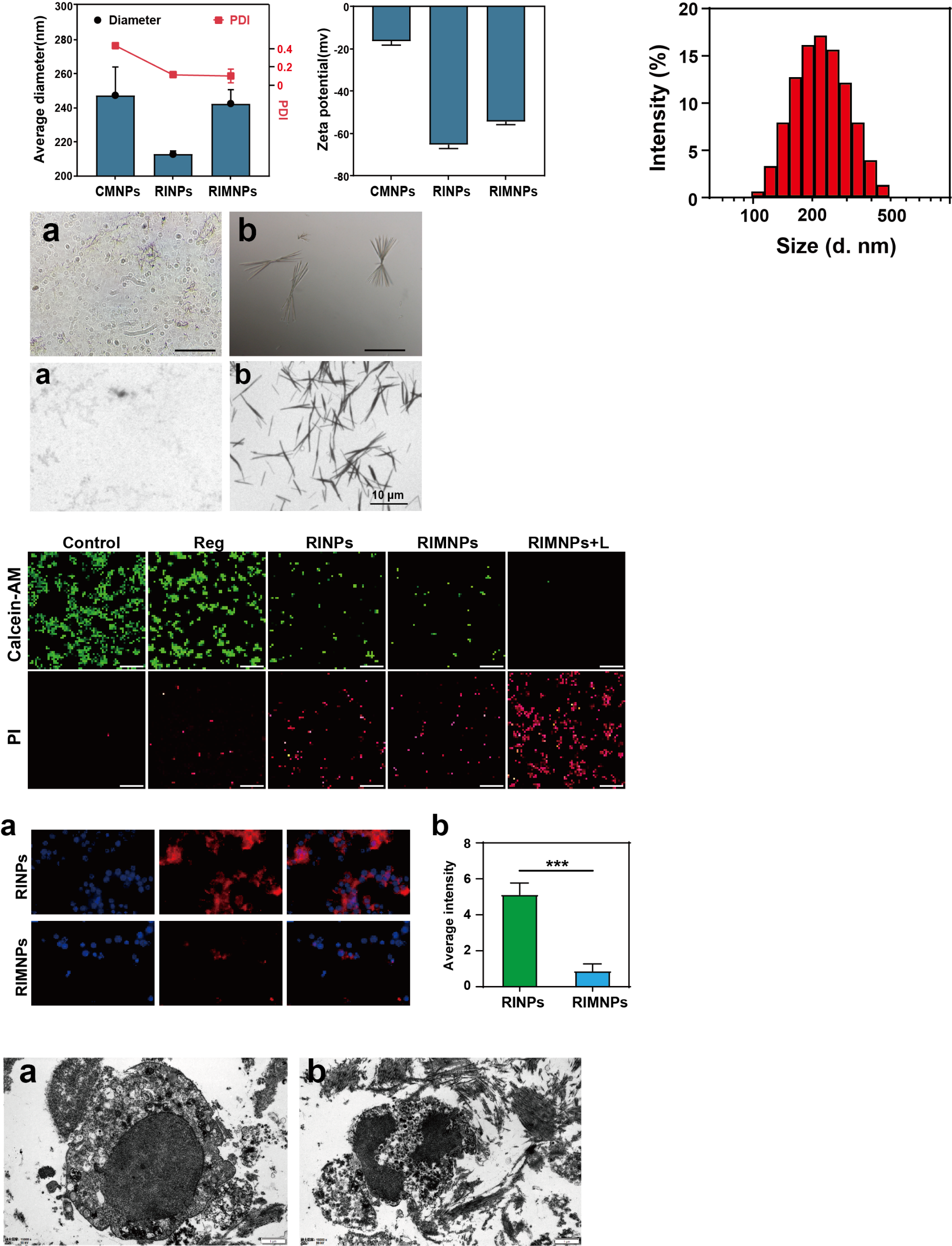


**Figure S17.** (a) Fluorescence images and (b) the relevant semi-quantitative analysis of RIMNPs after incubation with RAW 264.7 cells for 6 h (scale bar = 50 μm).


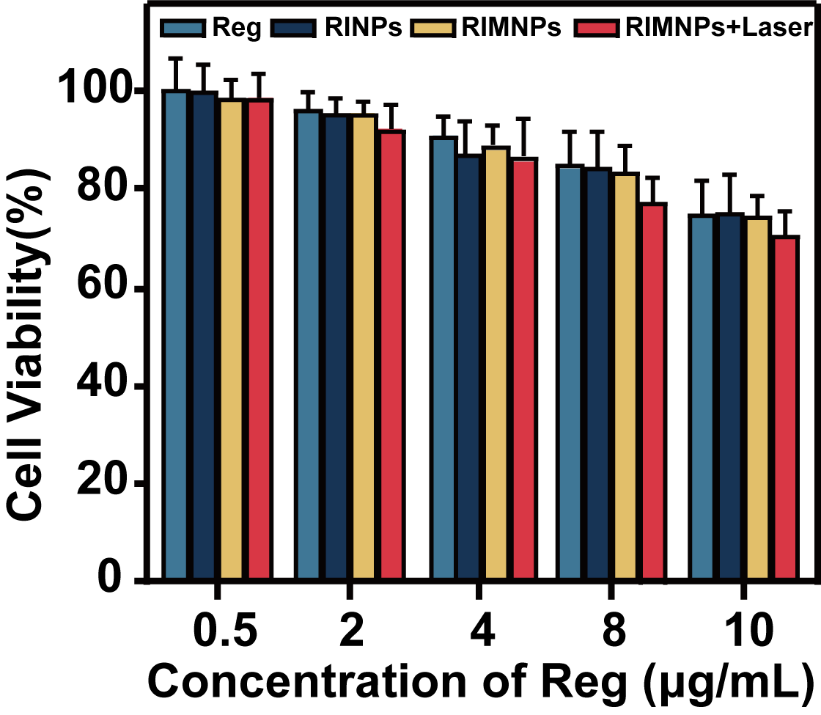


**Figure S18.** Cell viability of LO2 cells after being treated with Reg, RINPs, RIMNPs, and RIMNPs+Laser for 24 h with various concentrations of Reg (0.5, 2, 4, 8, 10 μg/mL).


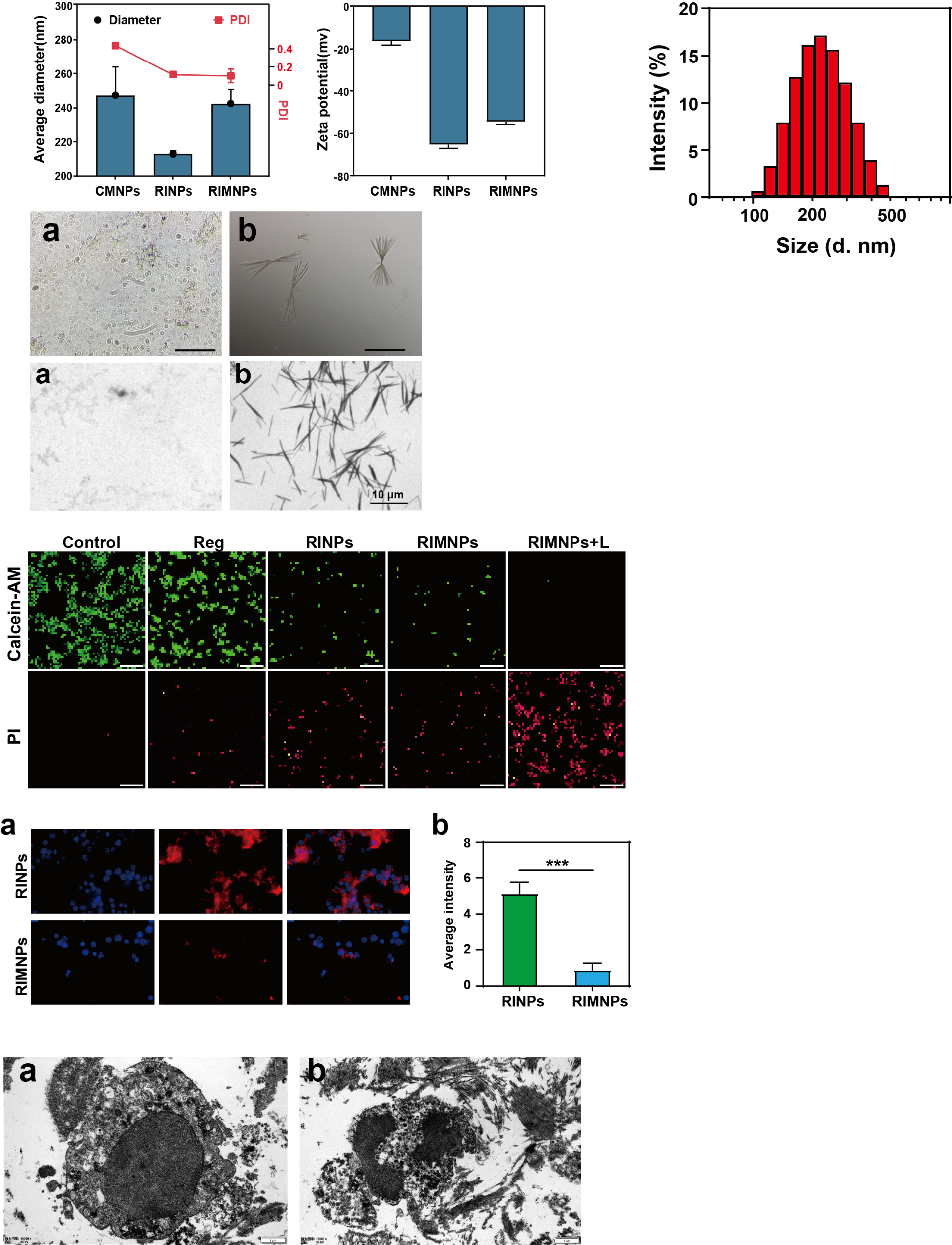


**Figure S19.**  Fluorescence images of HepG2 cells stained with Calcein-AM and PI with different treatments (Scale bar=20 μm).


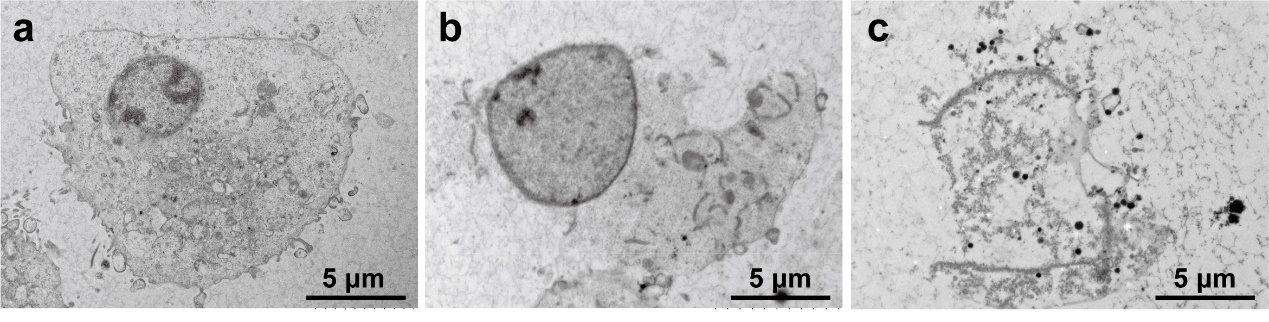


**Figure S20.** Bio-TEM of HepG2 cells incubated RIMNPs for 6 h (a) before and after irradiation with 808 nm laser for (b) 1 min and (c) 5 min respectively.


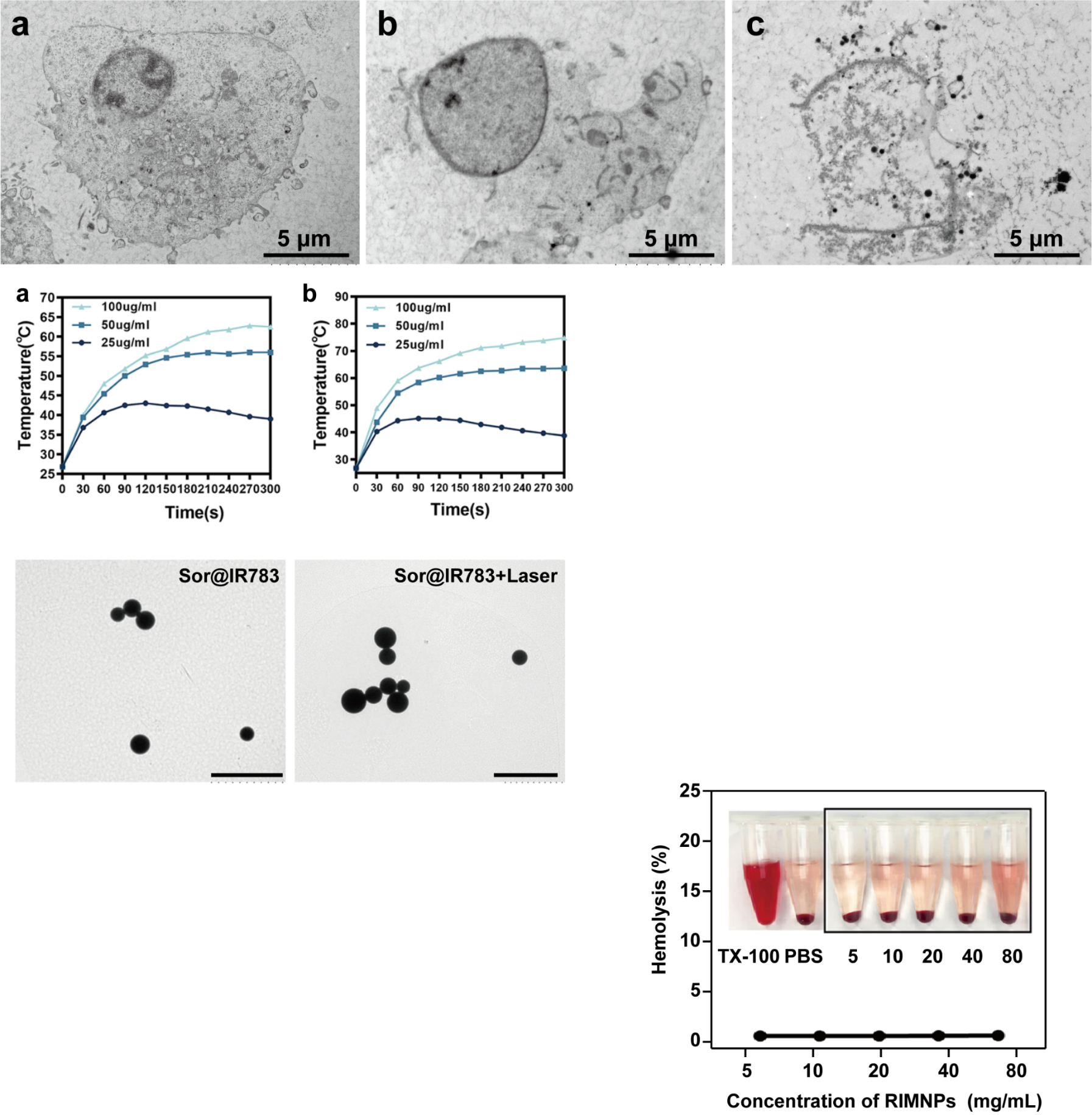


**Figure S21.** The hemolysis percentage and photographs of red blood cells (RBCs) incubated with RIMNPs at different concentrations. Positive (+) and negative (−) controls are the RBCs in water and PBS, respectively.


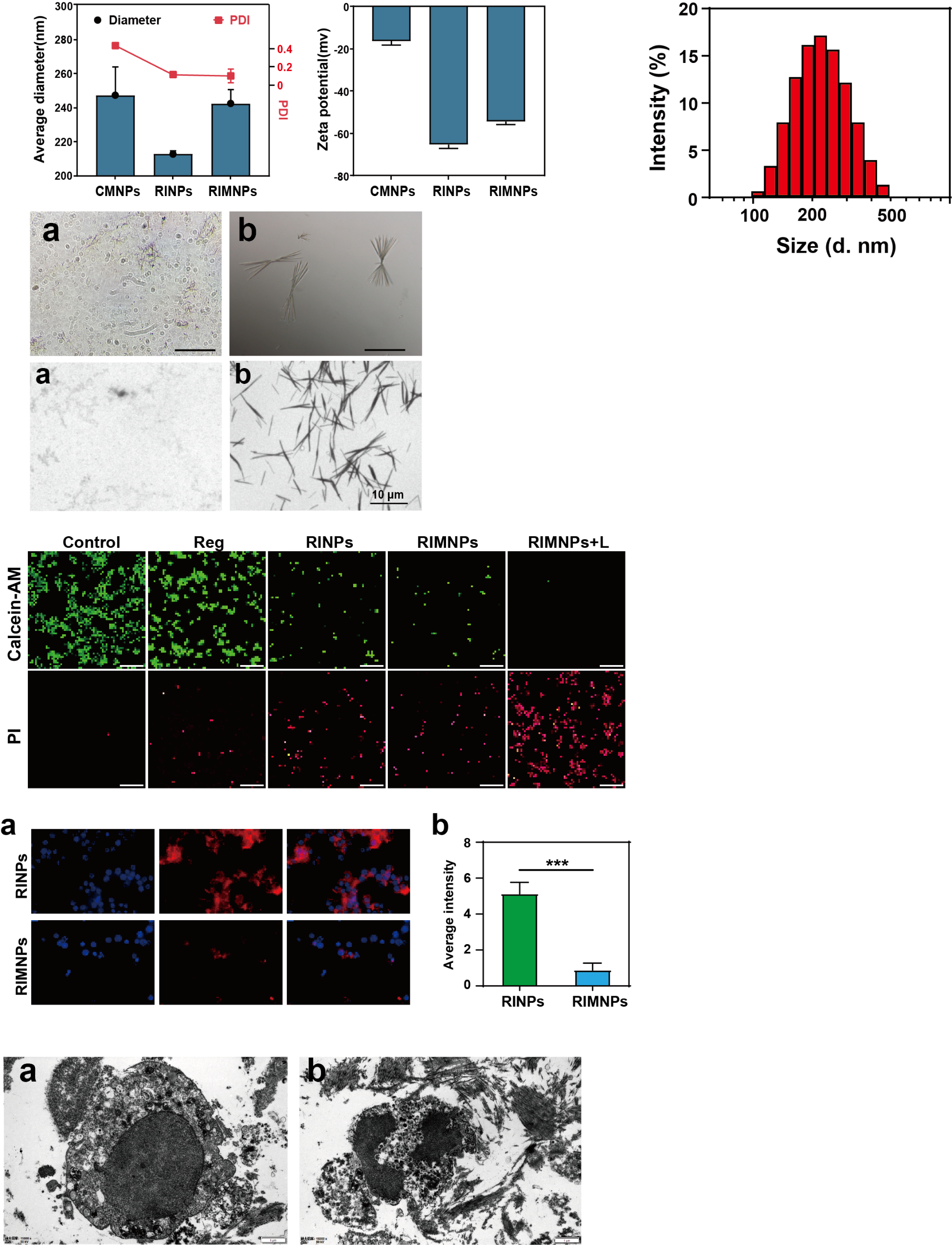


**Figure S22.** Bio-TEM images of HepG2 tumors intravenously administrated with RIMNPs before and after irradation with 808 lasers.


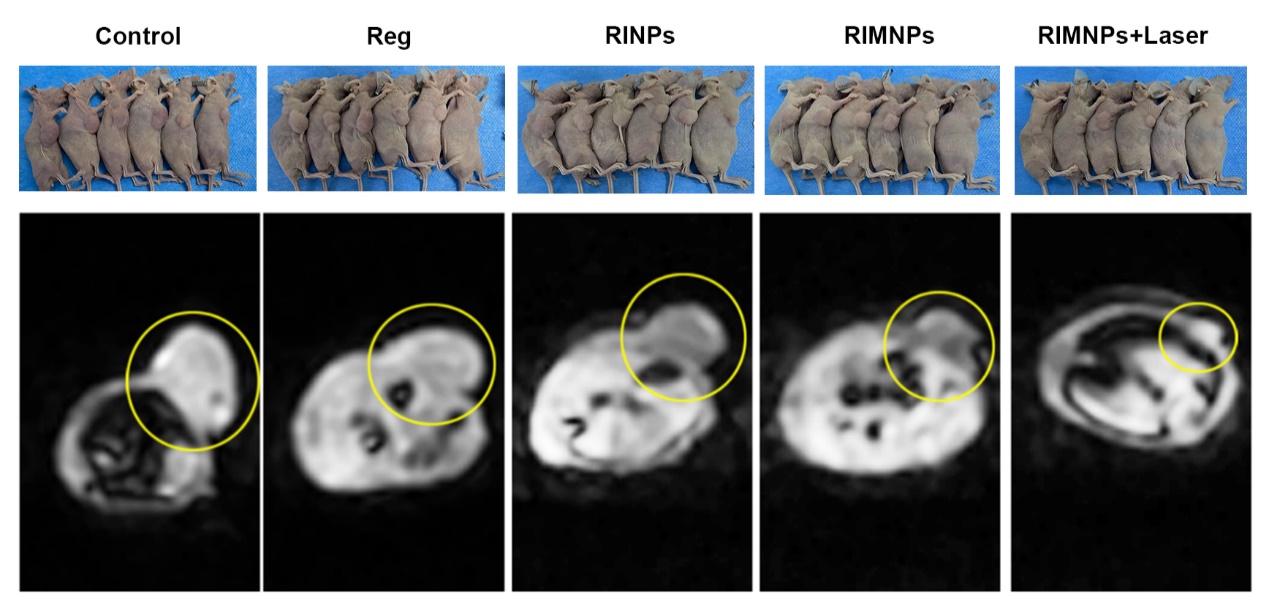


**Figure S23.** The images of tumor model mice at 14 days post-injection of PBS, Reg RINPs, RIMNPs, and CMNPs.


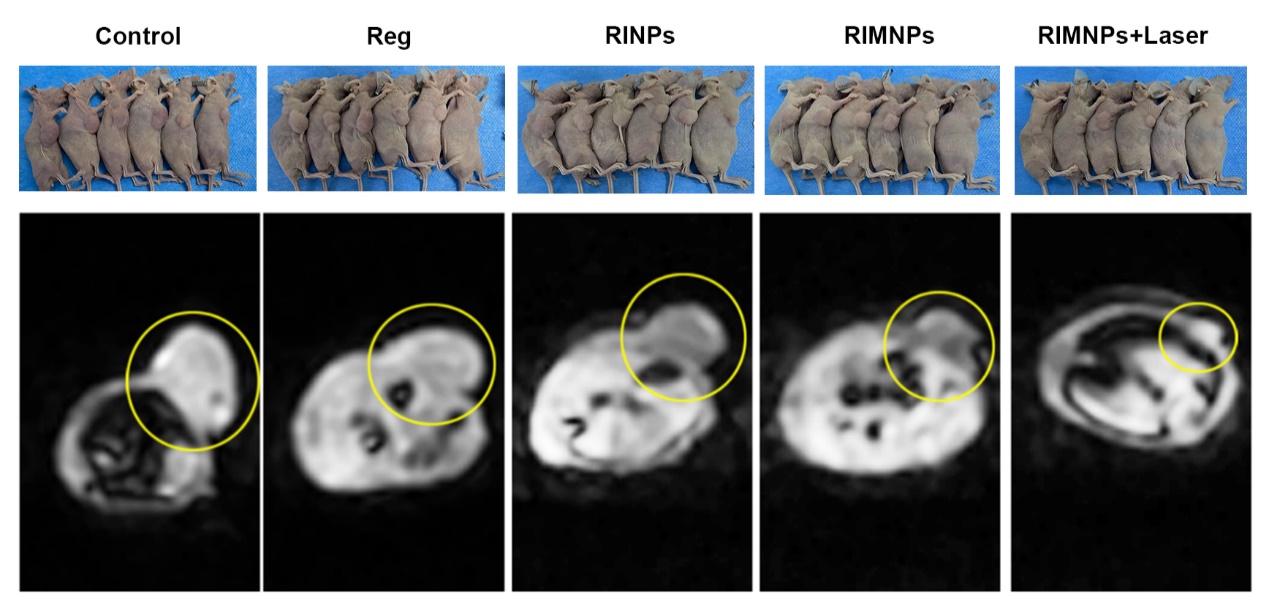


**Figure S24.** The MRI images at 14th day post-injection of PBS, Reg RINPs, RIMNPs, and CMNPs.
